# Decoding resistance in *Diutina catenulata* by validating clinically relevant Erg11/Fks1 mutations

**DOI:** 10.3389/fcimb.2026.1776442

**Published:** 2026-02-17

**Authors:** Wei Zhang, Na Wang, Xin-Fei Chen, Bao-Hua He, Meng Xiao, Ying-Chun Xu, Qi Li

**Affiliations:** 1 Hebei Key Laboratory of Pathogenic Mechanisms and Diagnosis & Treatment Technologies for Lung Microbiome, The First Affiliated Hospital of Hebei North University, Zhangjiakou, Hebei, China; 2 Hebei Key Laboratory of Pathogens and Epidemiology of Infectious Diseases, Hebei Provincial Center for Disease Control and Prevention, Shijiazhuang, Hebei, China; 3 Department of Laboratory Medicine, State Key Laboratory of Complex Severe and Rare Diseases, Peking Union Medical College Hospital, Chinese Academy of Medical Sciences, Beijing, China

**Keywords:** amino acid substitution, *Diutina catenulata*, ERG11, FKS1, gene mutation, molecular docking, recombinant plasmid

## Abstract

**Objective:**

To verify the causal relationship between specific mutations in the *ERG11* and *FKS1* genes and antifungal drug resistance in clinical isolates of *Diutina catenulata*.

**Methods:**

Recombinant plasmids expressing mutant alleles of *ERG11* (F126L, K143R) or *FKS1* (F621I, S1123G, I1348S, and the triple mutant S625L/S1123G/F1354L) were constructed and functionally validated in a *Saccharomyces cerevisiae* W303-1a model. Susceptibility testing was performed under different nutrient conditions (SD-Ura and YPD). Molecular docking analysis was conducted to elucidate the structural mechanisms of resistance.

**Results:**

Functional validation in *S. cerevisiae* confirmed that both *ERG11* and *FKS1* mutations conferred resistance in a nutrient-dependent manner. The *ERG11*-F126L mutation increased the fluconazole MIC by 21-fold in SD-Ura compared to YPD. *FKS1* mutations led to 1.4 to 2-fold increases in echinocandin MICs. Molecular docking revealed the mechanistic bases: *ERG11*-F126L expanded the ligand-binding cavity (ΔΔG +1.2 kcal/mol), *FKS1*-F621I disrupted hydrophobic interactions, and compound mutations synergistically perturbed ATP-binding domains.

**Conclusion:**

Specific mutations in *ERG11* (F126L, K143R) and *FKS1* (F621I and hotspot variants) are the primary drivers of the pronounced antifungal resistance observed in Chinese *D. catenulata* strains, with resistance phenotypes being modulated by nutrient availability.

## Graphical Abstract

**Figure:**
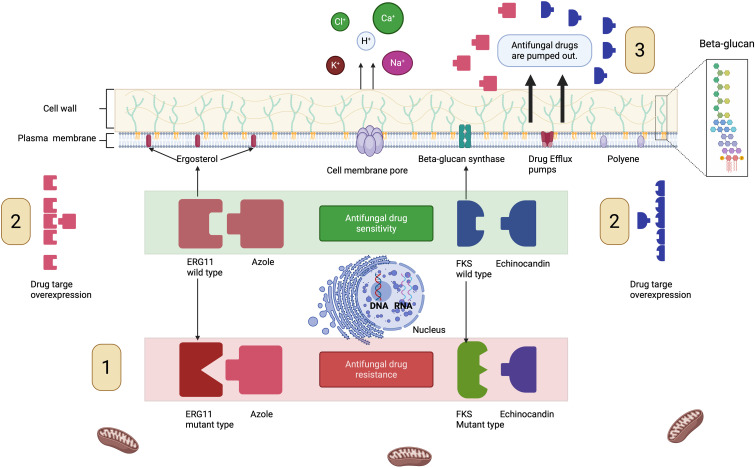
The resistance mechanisms of Candida species can be summarized into three primary categories: (1) Mutations in Drug-Binding Targets; (2) Overexpression of Drug Targets; (3) Efflux of Antifungal Drugs.

## 1 Introduction

Resistance of *Candida* species to azole and echinocandin antifungals has emerged as a critical public health challenge. Current research indicates that resistance is widespread in common species, such as *C. albicans* and *C. tropicalis*, as well as in rare strains, such as *C. auris* and *C. catenulata* (now reclassified as *Diutina catenulata*) (Pristov and Ghannoum, 2019; Arendrup and Patterson, 2017; Silva et al., 2012; Nourrisson et al., 2023; Díaz-García et al., 2022). Consequently, elucidating the mechanisms underlying resistance in *Candida* and related species has become a core focus in antifungal research. The known drug resistance mechanisms mainly include target protein mutations and overexpression of efflux pumps, but the specific roles of these mechanisms in *D. catenulata* are still unclear.

In this study, we used molecular docking techniques to analyze the spatial binding conformations of fluconazole and caspofungin with their target proteins (Erg11 and Fks1). Therefore, the aim of this study was to explore the basis for geographical differences in the antifungal resistance of *D. catenulata* through an integrated analysis of global susceptibility data and functional investigation of resistance mechanisms in clinical isolates from China.

## 2 Materials and methods

### 2.1 Literature review and ethical statement

The PubMed database was searched using the keywords “*Diutina catenulata*” OR “*Candida catenulata*,” and literature reporting human infections was screened. An analysis of the year of strain isolation, geographical distribution, patient sex, and sample type characteristics was conducted. Spatial distribution maps were generated based on the numbers of global and Chinese isolates. The Medical Ethics Committee of the First Affiliated Hospital of Hebei North University (Zhangjiakou, China) approved this study (No. K2025197) and exempted it from acquiring patient informed consent as it solely used *Diutina* isolates obtained from clinical samples without involving human genetic research.

### 2.2 Strain identification and antifungal drug sensitivity testing

Strain identification, antifungal susceptibility testing, and sequencing for key resistance genes of 11 Chinese isolates were described in our previous work (Chen et al., 2021). In general, the isolates were identified at the species level using matrix-assisted laser desorption/ionization time-of-flight mass spectrometry (Autobio ms1000; Autobio Diagnostics, Zhengzhou, China). Antifungal susceptibility testing against anidulafungin, micafungin, caspofungin, fluconazole, posaconazole, voriconazole, itraconazole, amphotericin B, and flucytosine was performed using the Sensititre YeastOne YO10 panel (Thermo Fisher Scientific, Waltham, MA, USA) according to the manufacturer’s instructions and Clinical and Laboratory Standards Institute (CLSI) guidelines. Briefly, *Diutina catenulata* isolates were inoculated onto Sabouraud dextrose agar medium and incubated at 35 °C for 24 h, after which single colonies were picked and suspended in sterile saline to achieve a 0.5 McFarland turbidity standard; 20 μL of this suspension was then added to an 11 mL broth tube, mixed thoroughly, and 100 μL aliquots were dispensed into each well of a 96-well plate, followed by incubation at 35 °C for 36 h before visually reading the MICs. MIC50 and MIC90 values were calculated from the collective MIC distributions using standard definitions, *C. parapsilosis* ATCC 22019 and *C. krusei* ATCC 6258 were used as CLSI-recommended QC strains to validate the performance of the broth microdilution method; these QC strains were not intended for biological comparison with *Diutina catenulata*.

### 2.3 Sequencing of target genes

Research has demonstrated that amino acid mutations in key target proteins are the primary drivers of antifungal resistance in *Candida* species, particularly to azoles via Erg11 (Cyp51) and to echinocandins via Fks subunits (Bédard et al., 2024; Jospe-Kaufman et al., 2024). To explore the link between such mutations and resistance in *D. catenulata*, we conducted Sanger sequencing of its *ERG11* and *FKS1* genes. First, we identified the homologous *ERG11* and *FKS1* proteins in *D. catenulata* by comparing published amino acid sequences (*ERG11*: accessions 3641571 and 1466526; *FKS1*: 3639844 and 856398) against the *D. catenulata* CBS 565 whole-genome sequence (PJEZ00000000.1) and performing gene annotation. Genomic DNA. was then extracted from the isolates, primers were designed and synthesized (with sequences detailed in **Supplementary Table 1**), and PCR. amplification was followed by Sanger sequencing. Finally, nucleotide and amino acid sequence variants were analyzed using CLC Sequence Viewer v.7.0 (QIAGEN Aarhus A/S, Aarhus, Denmark).

### 2.4 Recombinant plasmid construction

The target gene fragments (*ERG11*/*FKS1*) were amplified using PCR and ligated into the linearized pYES2/CT vector (double-digested with KpnI/XhoI). The ligation mixture was transformed into TOP10 competent cells through heat shock. Transformants were selected on Luria-Bertani agar plates containing ampicillin (37 °C, 16 h). Positive clones were verified using colony PCR, and recombinant plasmid sequences confirmed via Sanger sequencing.

### 2.5 Engineered strain resistance validation

The *S. cerevisiae* W303-1a strain was precultured in YPD medium (35 °C, 16 h). Competent cells were prepared using the Frozen EZ Yeast Transformation II Kit (Zymo Research, Irvine, CA, USA). Following plasmid transformation, the cells were plated onto SD-Ura agar and incubated at 30 °C for 48 h. Four single colonies were selected and cultured in SD-Ura liquid medium (30 °C, 220 rpm, 18 h). Cell suspensions were adjusted to the 0.5 McFarland standard in physiological saline. Suspensions were then spread onto YPD and SD-Ura plates, and MIC values determined using antimicrobial gradient diffusion strips (Liofilchem S.r.l., Roseto degli Abruzzi, Italy) (35 °C, 48 h). Solid medium was selected because it permits the discrimination of resistant subpopulations (Berman and Krysan, 2020).

### 2.6 Molecular docking simulations

In this study, fluconazole and caspofungin showed a more pronounced reduction in activity against 11 Chinese clinical isolates of *Diutina catenulata* than other drugs in their respective classes. Therefore, we performed molecular docking simulations of these two agents with their corresponding target proteins to investigate potential drug–target interactions that may underlie the decreased susceptibility. The three-dimensional structures of fluconazole (CAS 86386-73-4) and caspofungin (CAS 162808-62-0) were retrieved in SDF format from the PubChem database. Energy minimization (Root Mean Square gradient threshold: 0.001) was performed using ChemBio3D Ultra v.14.0, outputting mol2 files. AutoDockTools v.1.5.6 (Scripps Research, La Jolla, San Diego, California, USA) was used to add hydrogen atoms, calculate charges, assign rotatable bonds, and convert files to the pdbqt format. Protein structures were predicted using AlphaFold. Water molecules and ligands were removed using PyMOL v.2.3.0. (Schrödinger, New Jersey, USA) Receptor preparation followed the same protocol as that for ligands using AutoDockTools. Molecular docking was performed using AutoDock Vina v.1.1.2. Interaction patterns were visualized in PyMOL, and final images generated using Adobe Illustrator.

### 2.7 AI tool statement

During the preparation of this work, the authors used DeepSeek-R1 to improve the readability of the manuscript. After using this tool, the authors reviewed and edited the content as needed and take full responsibility for the content of the publication.

## 3 Results

### 3.1 Antifungal susceptibility profile and epidemiological of *Diutina catenulata*

To assess the geographical variations in antifungal resistance reported in the literature, we compiled an integrated dataset of susceptibility profiles. This dataset comprised 65 *D. catenulata* isolates—including 11 clinical isolates from China (current study), 45 from France [literature-derived (Nourrisson et al., 2023)], and 9 from Brazil [literature-derived (Almeida-Paes et al., 2024). The *in vitro* susceptibility of 11 Chinese *D. catenulata* isolates (current study) to nine antifungal agents revealed significant variations in MICs. Among azole antifungals, fluconazole (A) exhibited the highest MIC. values (4–256 µg/mL), with 63.6% (7/11) of isolates meeting the resistance breakpoint (≥32 µg/mL). Voriconazole (B) demonstrated an MIC. range of 0.06–4 µg/mL (27.3% ≥1 µg/mL), while itraconazole (C) and posaconazole (D) showed consistently lower MICs. (0.03–0.12 µg/mL). 5-Fluorouracil (E) exhibited MIC. values spanning 0.06–0.12 µg/mL. Amphotericin B (F) maintained uniformly low MICs (0.125–1 µg/mL) across all isolates. Notably, caspofungin (G), micafungin (H), and anidulafungin (I) demonstrated elevated MICs (≥1 µg/mL) in 81.8% (9/11), 45.5% (5/11), and 54.5% (6/11) of isolates, respectively (**Supplementary Figure 1**).

For fluconazole, the Chinese isolates showed MIC_50_/MIC_90_ values 2,000-/2,048-fold higher than those of the French isolates and 8-/16-fold higher than those of the Brazilian isolates. Reversed susceptibility was observed for voriconazole, with French isolates exhibiting MIC_50_/MIC_90_ values 12-/48-fold higher than those of Chinese isolates and 47-/384-fold higher than those of Brazilian isolates. Posaconazole and itraconazole exhibited consistently potent activity against all isolates (MIC_50_/MIC_90_ ≤0.12 μg/mL). Amphotericin B showed uniform activity across regions at MIC_50_ levels ≤0.5 μg/mL, with slight MIC_90_ variations noted: China, 1 μg/mL; France, 0.75 μg/mL; and Brazil, 0.5 μg/mL. Micafungin exhibited stark cross-country resistance disparities. Chinese isolates demonstrated MIC_50_/MIC_90_ values 43-fold/250-fold higher than those of French isolates and 32-fold/64-fold higher than those of Brazilian isolates. Similar trends were observed for caspofungin, although these were less pronounced.

Chinese and Brazilian (Almeida-Paes et al., 2024) isolates originated exclusively from human clinical specimens. French isolates were derived from diverse reservoirs, including human hosts (clinical), animals, food products, and environmental sources (Nourrisson et al., 2023). Nucleotide sequences of *ERG11/FKS1* genes, corresponding Erg11*/*Fks1 protein amino acid sequences, and the plasmid (pYES2/CT) genetic sequence are provided in **Supplementary File 1**.

**Figure 1:**
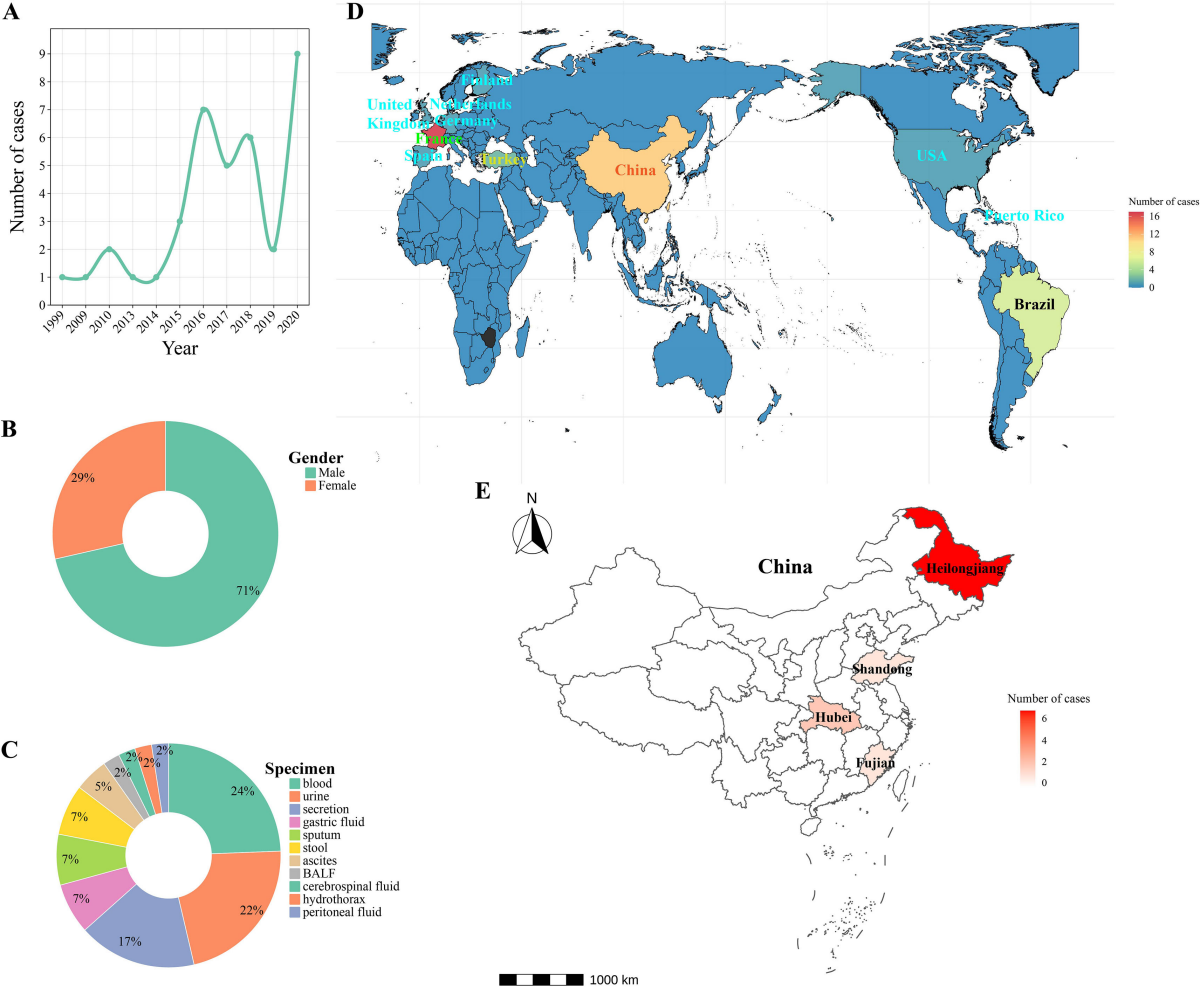
Global epidemiology of *Diutina catenulata* infections (1999–2020) (Radosavljevic et al., 1999; Ha et al., 2018; Çakir et al., 2019; Nourrisson et al., 2023; Chen et al., 2021). **(A)** Temporal trend showing increasing case reports. **(B)** Gender distribution. **(C)** Clinical specimen sources. **(D)** Global case distribution. **(E)** Geographic distribution within China, highlighting Heilongjiang Province (current study, the rest are from literature). BALF, bronchoalveolar lavage fluid.

Among these 65 strains, 38 were associated with human infections, with the remainder originating from veterinary and environmental sources. Analysis of the 38 infection cases revealed a male predominance (male-to-female ratio = 2.5:1; males accounted for 71%). The primary specimen types were blood (24%), urine (22%), and wound exudates (17%). Within China, the 11 reported cases were distributed across Heilongjiang (64%), Hubei (18%), Shandong (9%), and Fujian (9%) provinces (**Figure 1**). Key susceptibility metrics (MIC50/MIC90, μg/mL), which reveal geographical variations, are summarized in **Table 1**.

**Table 1:** MIC_50_/MIC_90_ values of seven antifungal agents against *Diutina catenulata* clinical isolates from China (current study), France (literature), and Brazil (literature).

Antifungal	MIC_50_ (µg/mL)			MIC_90_ (µg/mL)		
	^a^China	^b^France	^c^Brazil	^a^China	^b^France	^c^Brazil
Fluconazole	64	0.032	8	256	0.125	16
Voriconazole	0.25	3	0.063	1	48	0.125
Posaconazole	0.06	0.012	0.063	0.12	0.023	0.063
Itraconazole	0.12	0.012	0.015	0.12	0.023	0.031
Amphotericin B	0.5	0.5	0.25	1	0.75	0.5
Caspofungin	>8	0.19	ND	>8	0.38	ND
Micafungin	1	0.023	0.031	>8	0.032	0.125

MIC, minimum inhibitory concentration; ND, no data.

a The 11 clinical isolates of *Diutina catenulata* analyzed in this study were obtained from 11 infected patients in China. For comparative analysis, additional susceptibility data from French and Brazilian isolates were incorporated from the literature.

b Reference (Nourrisson et al., 2023): Forty-five *Diutina catenulata* isolates were obtained from diverse. sources, including human hosts (clinical), animals, food products, and environmental reservoirs.

c Reference (Almeida-Paes et al., 2024): Nine *Diutina catenulata* isolates were cultured from clinical specimens collected from six hospitalized patients.

### 3.2 Correlation analysis between amino acid mutations and MIC values

Among the 11 Chinese *D. catenulata* isolates, amino acid substitutions in Erg11 and Fks1 proteins correlated with elevated antifungal MICs. Panel A demonstrates Erg11 mutations: the F126L substitution occurred in isolates 10H1065, 16H4241, 16HLJ6019, 17HLJ6024, and 17HLJ6025, all exhibiting fluconazole MICs of 32–256 µg/mL, while the K143R mutation in strains 10H1051 and 18QH369 corresponded to fluconazole MICs of 64 and 256 µg/mL, respectively. Panels B-D reveal Fks1 mutations: isolates with F621I (10H1065, 16H4241) showed caspofungin MICs of 8 µg/mL; strain 17TJ970 bearing a triple mutation (S625L+S1123G+F1354L) exhibited MIC 8 µg/mL; isolates carrying S1123G (13TJ359, 15FJ457) demonstrated MICs of 4 and 1 µg/mL; and isolates with I1348S substitution (16HLJ6019, 17HLJ6024, 17HLJ6025) all displayed caspofungin MICs of 8 µg/mL (**Supplementary Figure 2**).

### 3.3 Construction of recombinant plasmids

**Figure 2:**
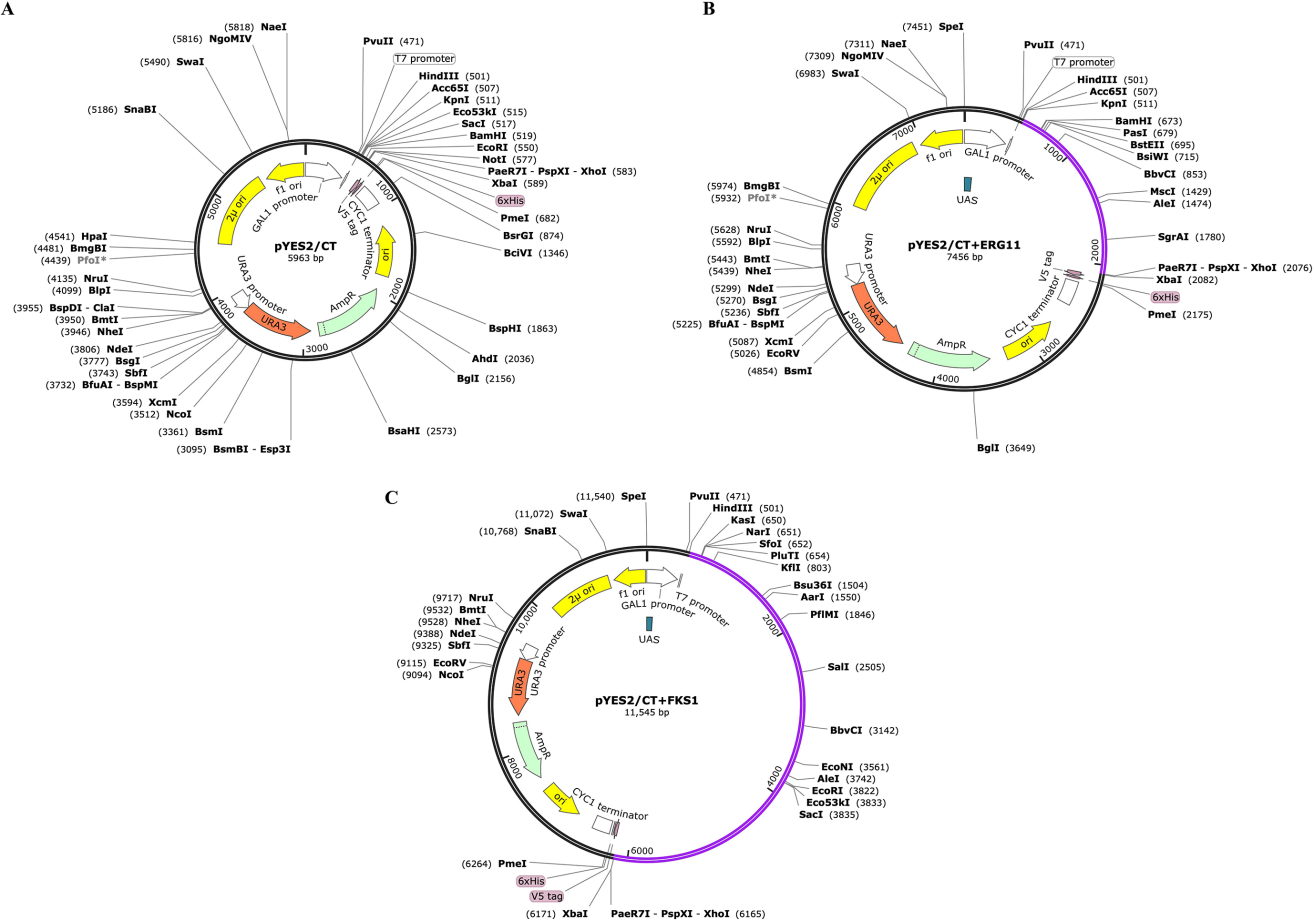
Plasmid gene map. **(A)** Original plasmid pYES2/CT. **(B)** Recombinant plasmid pYES2/CT+ERG11 gene. **(C)** Recombinant plasmid pYES2/CT+FKS1 gene.

**Figure 3:**
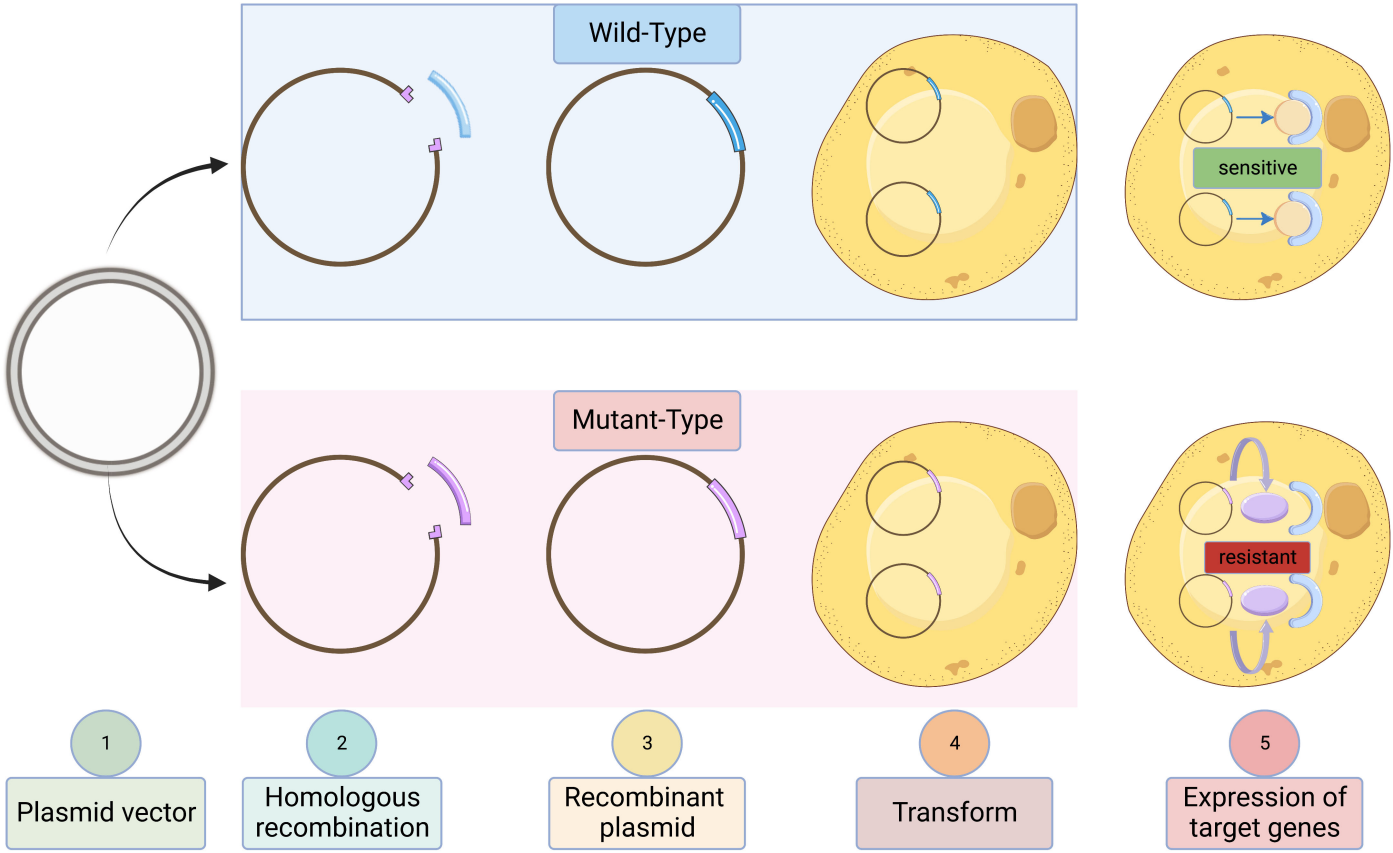
Validation of antifungal resistance in engineered *Saccharomyces cerevisiae* W303-1a strains. Schematic representation contrasting the wild-type strain (antifungal-sensitive) with the recombinant mutant strain expressing target genes (antifungal-resistant).

Recombinant plasmids were successfully constructed from the pYES2/CT vector for expression in *S. cerevisiae* W303-1a (**Figures 2**, **3**). For the *ERG11* recombinant plasmid (7,456 bp), a 1,563-bp fragment encoding 520 amino acids was inserted via the KpnI/XbaI restriction sites. Similarly, the *FKS1* recombinant plasmid (11,545 bp) was constructed by inserting a 5,652-bp fragment encoding 1,883 amino acids. Expression was induced by galactose. Vector maps were verified through visualization using SnapGene software.

### 3.4 Confirming ERG11-mediated resistance causality

**Figure 4:**
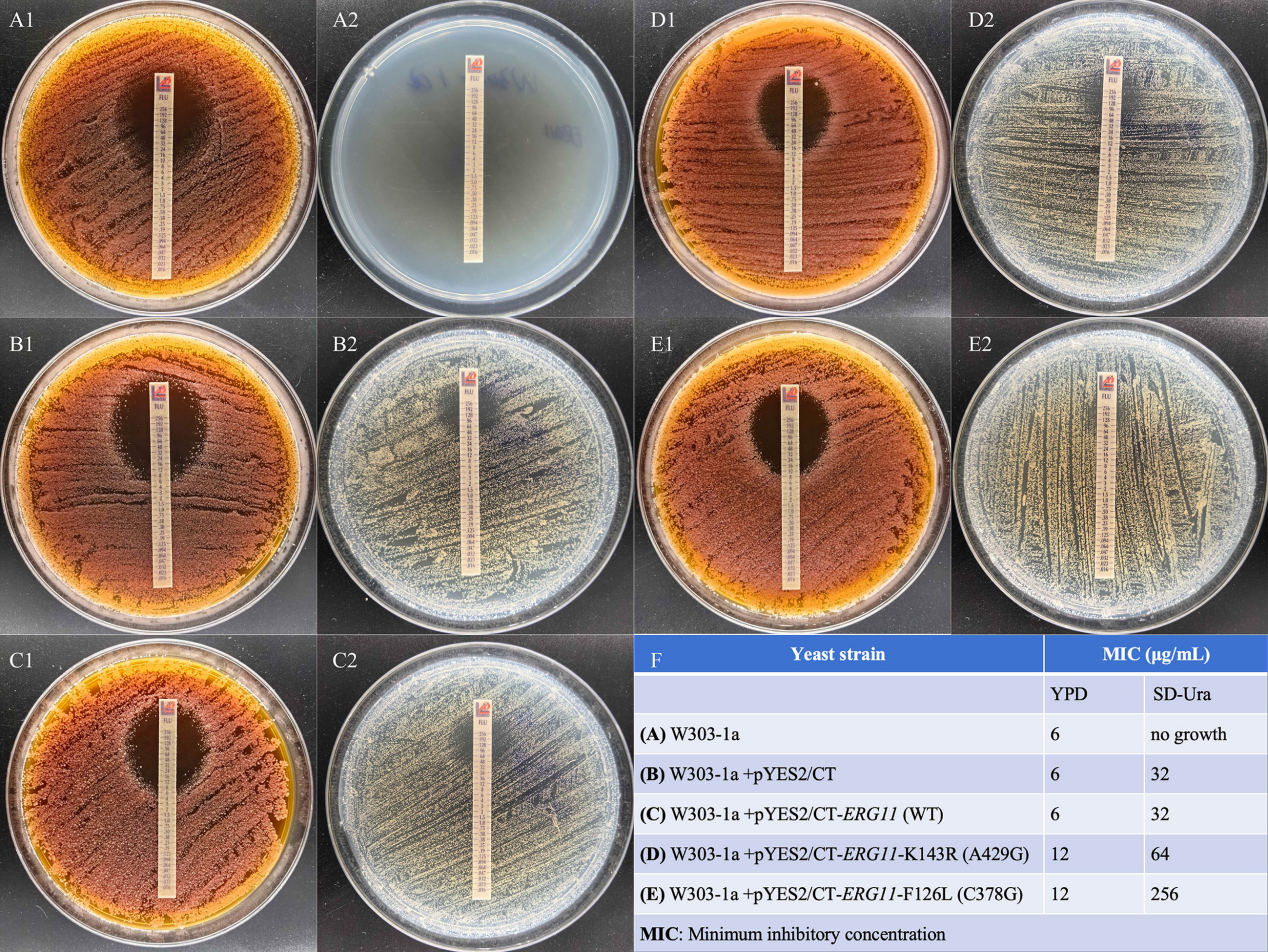
Fluconazole minimum inhibitory concentration (MIC) results. **(A–E)** Antifungal susceptibility testing on (1) YPD agar and (2) SD-Ura agar plates. **(F)** Summary table of MIC values.

Fluconazole susceptibility profiling in *S. cerevisiae* revealed mutation- and medium-dependent resistance patterns (**Figure 4**). In YPD medium, strains carrying *ERG11* mutations showed limited resistance (MICs: W303-1a parental, 6 μg/mL; empty vector/pYES2/CT, 6 μg/mL; wild-type Erg11, 6 μg/mL; K143R mutant, 12 μg/mL; F126L mutant, 12 μg/mL). In contrast, in SD-Ura medium, resistance amplified considerably: F126L mutants exhibited 8-fold higher MICs (256 μg/mL) than the wild-type Erg11 controls (32 μg/mL), whereas K143R mutants showed 2-fold higher resistance (64 μg/mL), confirming that nutrient stress potentiates Erg11 mutation-mediated azole resistance.

### 3.5 Confirming FKS1-mediated resistance causality

**Figure 5:**
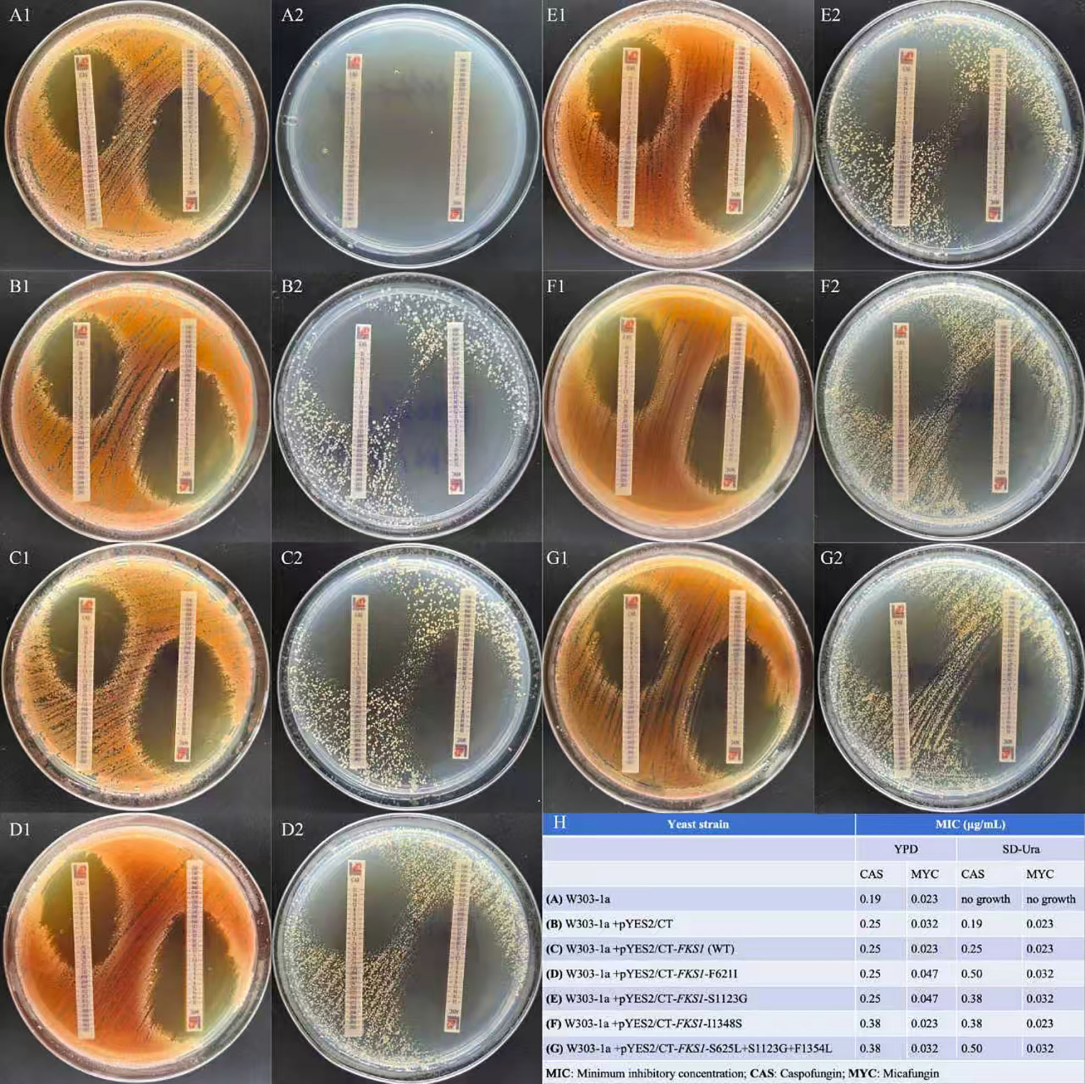
Caspofungin and micafungin minimum inhibitory concentration (MIC) results. **(A–G)** Antifungal susceptibility testing on (1) YPD agar and (2) SD-Ura agar plates. **(H)** Summary table of MIC values.

Both echinocandins demonstrated identical MIC elevations across *FKS1*-mutant transformants in YPD medium: F621I and S1123G mutants retained the wild-type susceptibility (1-fold MIC), whereas I1348S and S625L/S1123G/F1354L mutants exhibited moderate resistance (1.5-fold MIC increase) (**Figure 5**). Caspofungin susceptibility uniformly decreased in all mutants grown in SD-Ura medium (1.5–2-fold MIC increase vs. wild-type). Micafungin showed differential resistance, with mutants F621I, S1123G, and S625L/S1123G/F1354L displaying a modest MIC elevation (1.4-fold), whereas the I1348S mutant maintained the wild-type susceptibility. The parental W303-1a strain was nonviable in SD-Ura medium when grown without the pYES2/CT plasmid.

### 3.6 Molecular docking and mechanism analysis

**Figure 6:**
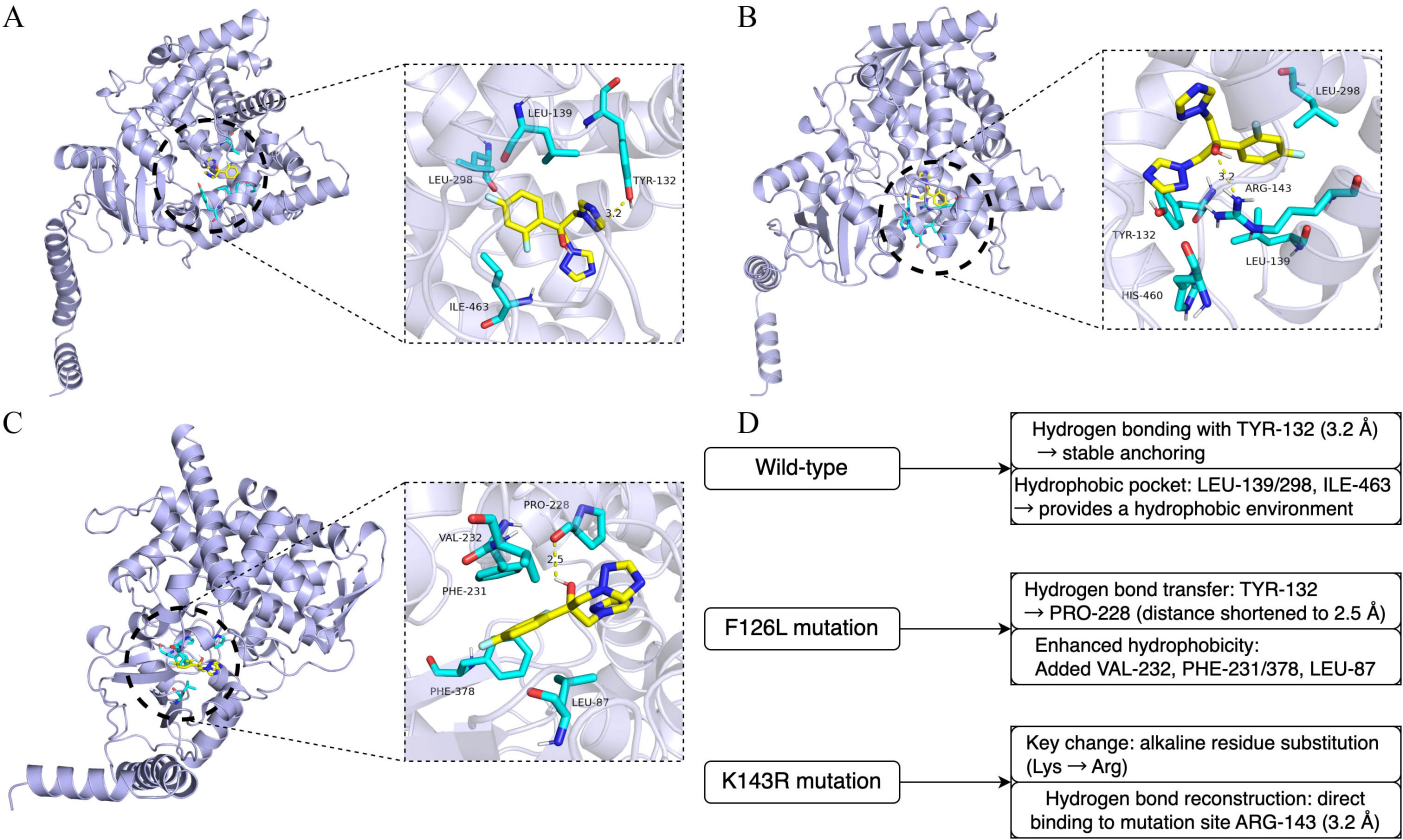
Molecular docking simulation of fluconazole with target protein Erg11. **(A)** Wild-type Erg11. **(B)** F126L amino acid mutation. **(C)** K143R amino acid mutation. **(D)** Explanatory diagram of **(A–C)**.

**Figure 7:**
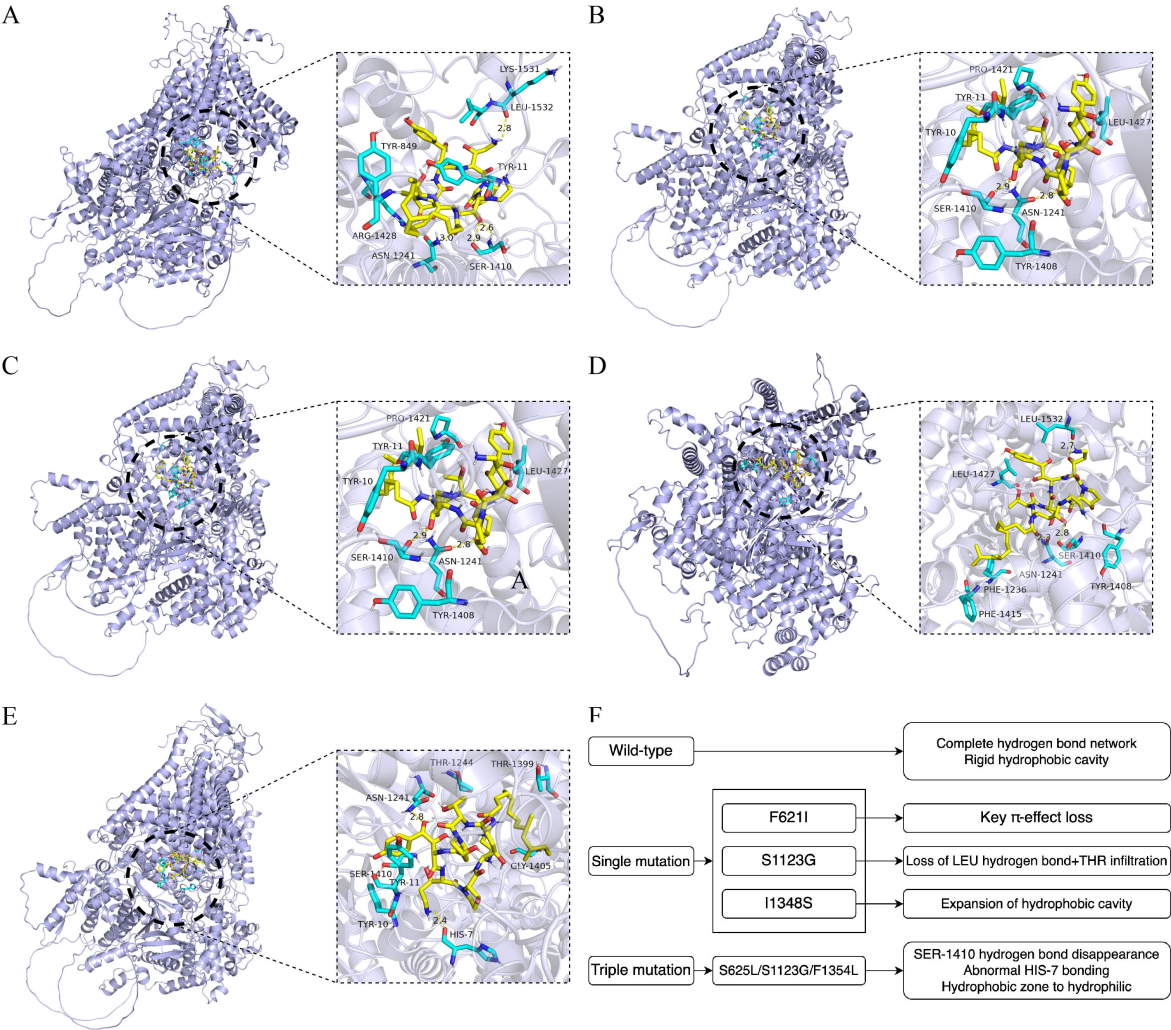
Molecular docking simulation of caspofungin with target protein Fks1. **(A)** Wild-type Fks1. **(B)** F621I amino acid mutation. **(C)** S1123G amino acid mutation. **(D)** I1348S amino acid mutation. **(E)** S625L/S1123G/F1354L triple mutation. **(F)** Explanatory diagram of **(A–E)**.

Molecular docking and mechanism analysis of fluconazole with Erg11 (**Figure 6**) and Fks1 (**Figure 7**) protein variants revealed notable differences when compared with the wild-types (**Supplementary File 1**). The F126L mutation in Erg11 caused an expansion in the ligand-binding cavity (volume increased by 23%) and loss of hydrophobic contacts, resulting in a binding free energy change (BINDING FREE ENERGY CHANGE) of +1.2 kcal/mol [this was partially reversible by exposure to high-dose fluconazole (Moreau et al., 2024) (Benjamin et al., 2021)]. The K143R mutation in Erg11 disrupted the hydrogen-bonding network [partially compensated by His381 (Alsulaimany et al., 2025)], causing binding pocket deformation with a BINDING FREE ENERGY CHANGE of +0.6 kcal/mol.

The F621I mutant in Fks1 caused a 30% loss in hydrophobic volume, which disrupted π–π stacking [key mutation in Hotspot region 1 (Chen et al., 2021)]. The S1123G mutant in Fks1 resulted in a loss of hydrogen-bonding ability, with conformational perturbation observed. The Fks1 mutant, I1348S, reduced the side-chain volume by 30 Å^3^, which induced a polar repulsion effect (characteristic of Hotspot region 2). The compound mutation (S625L/S1123G/F1345L) synergistically perturbed conformation of the ATP-binding domain, which reduced substrate affinity.

## 4 Discussion

### 4.1 Systemic fungal infections pose significant clinical challenges

Advances in identification technologies have led to the discovery of novel *Candida* and related species. Notably, *C. auris* has caused outbreaks of lethal infections in more than 30 countries because of its high invasiveness, multidrug resistance, and immune evasion capabilities. Fluconazole resistance in *C. parapsilosis* can reach 26.7% (Ünal et al., 2024), with resistance profiles exhibiting dependence on sample type—blood isolates show significantly higher fluconazole resistance rates compared with other sample types (9.1% versus 8.2%; P &gt; 0.05), whereas echinocandin resistance shows an opposite distribution pattern (Díaz-García et al., 2022). In contrast, *D. catenulata* primarily inhabits the avian gut and is used in cheese production; human infections are exceptionally rare. The current literature reports only 38 documented cases of *D. catenulata* infections in humans globally (Radosavljevic et al., 1999; Ha et al., 2018; Çakir et al., 2019; Quindós et al., 2022; Nourrisson et al., 2023; Chen et al., 2021). In the present study, only 11 isolates collected over an 8-year period (2010–2018) from clinical institutions in China were identified. Their dispersed geographical distribution, spanning four distinct provinces in the north and south of China, suggests a low pathogenic potential, likely positioning *D. catenulata* as an opportunistic pathogen.

Despite its low prevalence in invasive infections, *D. catenulata* exhibits a notably high antifungal resistance profile. Our study revealed that clinical isolates from China exhibited a multidrug-resistant phenotype; fluconazole resistance reached 63.6% (7/11) and caspofungin resistance increased to 81.8% (9/11), which significantly surpassed that typically observed in common pathogens, such as the resistance rate of *Candida albicans* to fluconazole is only 9.6%. This paradoxical combination of rare pathogens with high resistance suggests that they may have undergone adaptive evolution under drug selection pressure, underscoring the urgent need for resistance surveillance and a mechanistic understanding of public health.

### 4.2 Escalating resistance amidst limited therapeutic options

Key mechanisms underlying azole resistance include target protein modifications (e.g., Erg11 mutations that reduce drug affinity), gene dosage effects (e.g., increased *Erg11* copy numbers leading to target overexpression), and efflux pump overexpression. Critical residue mutations in the Erg11 protein of *C. albicans*, including A114S and Y257H, have been confirmed to drive fluconazole resistance (Esfahani et al., 2022). Gain-of-function mutations in the transcription factor Pdr1 in *C. glabrata* enhance azole resistance (Tian et al., 2020). Echinocandin resistance is primarily attributed to mutations in *FKS* genes, which encode the drug target, β-1,3-glucan synthase. Specific residues in Fks1 (S629P) and Fks2 (S663F) of *C. glabrata* have been validated as resistance hotspots (Shields et al., 2019).

Currently available antifungal drug choices are severely limited, whereas challenges with fungal resistance to drugs are intensifying globally. More concerningly, treatment failure occurs even with drug-sensitive strains, which are strongly linked to resistance that is evolutionarily driven by environmental pressures, such as climate change and antimicrobial exposure. Primary fungal resistance mechanisms include altered drug-target interactions, efflux pump-mediated reduction of intracellular drug concentrations, and biofilm barrier effects.

The present study focused on target gene mutation-induced resistance and confirmed that the Erg11 mutations, K143R and F126L, confer fluconazole resistance in *D. catenulata*, elevating the MIC by 8-fold in clinical isolates. Using the *S. cerevisiae* heterologous expression system, we validated that the K143R mutation increased the fluconazole MIC by 2-fold, whereas the F126L mutation increased it by 8-fold (in SD-Ura medium). These findings align with those of previous research on *C. albicans*, which indicate that approximately 33% of Erg11 mutants develop azole resistance, with 88% showing cross-resistance (Bédard et al., 2024).

### 4.3 Molecular mechanisms

**Figure 8:**
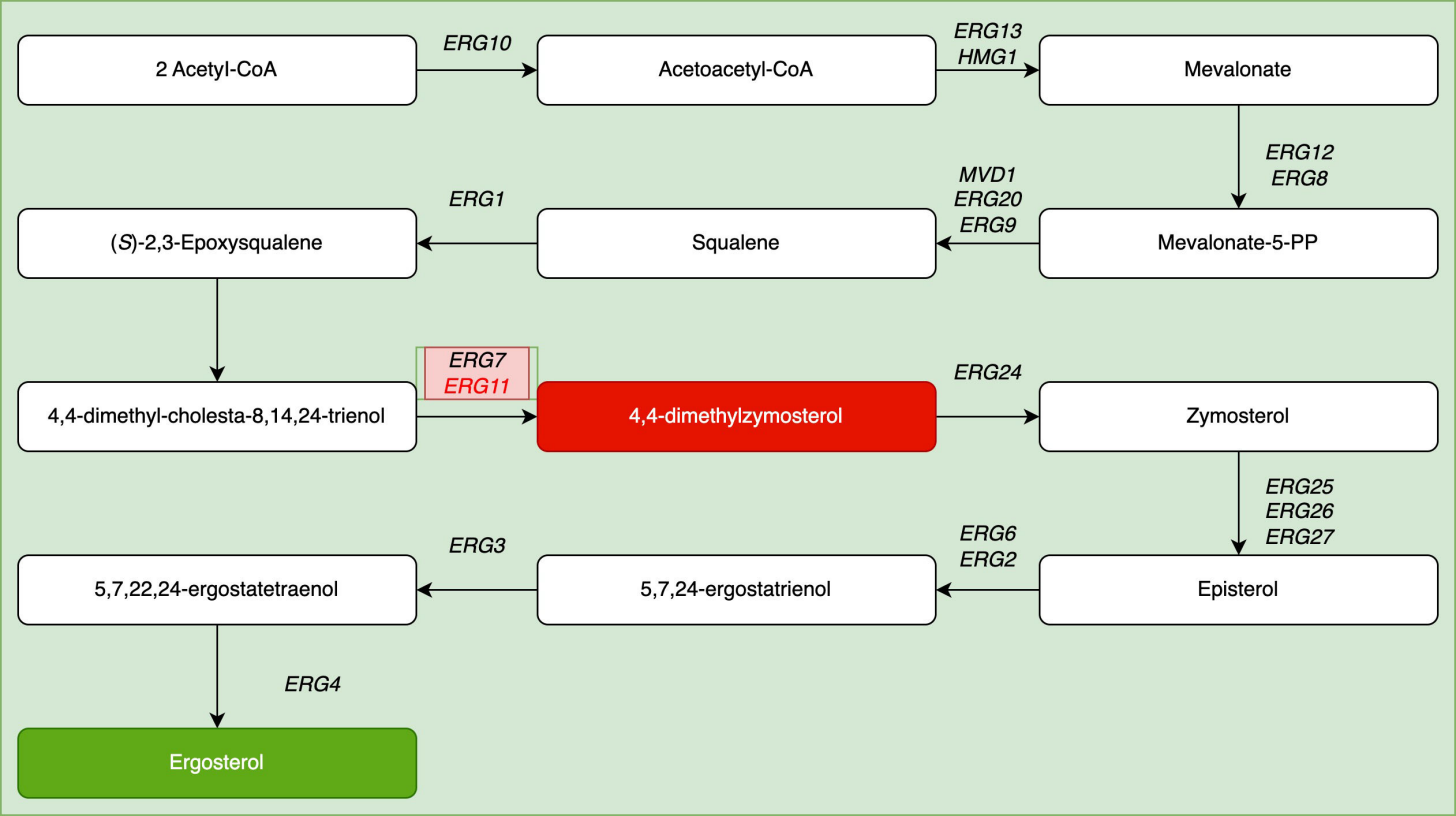
Schematic representation of the ergosterol biosynthesis pathway (Zhang et al., 2015).

*ERG11* encodes sterol 14α-demethylase, which catalyzes the essential conversion of lanosterol to ergosterol (**Figure 8**). Azoles inhibit this enzyme, leading to the toxic accumulation of 14α-methylsterols and fungicidal activity. Similarly, a G476S mutation in *Plenodomus* increased the fluconazole MIC by 7.3-fold (King et al., 2025), and mutations (VF125AL and K177R/N335S/E343D) in *C. auris* strains isolated in South Africa resulted in high resistance rates (96.3%, MIC >32 μg/mL) (Kekana et al., 2023).

*FKS1*, encoding the membrane-integrated β-1,3-glucan synthase, is the primary target of echinocandins and the new agent, ibrexafungerp. In clinical *D. catenulata* isolates, the hotspot mutation, F621I, increased the caspofungin and micafungin MICs by 2-fold and 4-fold, respectively. Engineered Fks1 containing F621I in yeast showed a corresponding 2-fold increase in the MIC of caspofungin. This parallels the findings observed for *C. glabrata*, where S629P/S663P mutations caused caspofungin MICs to reach 8 μg/mL (Beyda et al., 2014), indicating the presence of conserved mechanisms. Notably, echinocandin resistance mutations were predominantly clustered within the transmembrane domains of Fks1 (TM5–6 and TM8) (Hu et al., 2023). Amino acid substitutions in these mutation hotspots reduce drug sensitivity via steric hindrance (Hu et al., 2023; Perlin, 2015).

### 4.4 Study limitations and future directions

Although CLSI QC strains were included to ensure methodological validity, they are phylogenetically distant from D. catenulata and have distinct antifungal susceptibility profiles; therefore, QC results confirm assay performance but do not imply biological comparability or inform intrinsic susceptibility of *D. catenulata.* A limitation of this study is that functional validation was performed using heterologous expression in *S. cerevisiae*, which may not capture species-specific regulation, cell wall or membrane composition, or epistatic interactions present in *D. catenulata*. In addition, SD-Ura represents an *in vitro* nutrient-limited stress condition and should not be interpreted as a direct proxy for *in vivo* host environments. Thus, while these experiments support a causal contribution of specific *ERG11* and *FKS1* mutations to altered susceptibility, the magnitude of resistance and its clinical impact require confirmation in the native background and *in vivo* models. Although CDC ECOFF thresholds confirmed MIC elevations, undefined clinical breakpoints for *D. catenulata* and static molecular docking data limit translational relevance. Research on resistance mechanisms involving efflux pumps and gene copy number variations was not performed. Future work should include larger, regionally diverse sampling with whole-genome sequencing to track *D. catenulata* epidemiology and resistance evolution, alongside in-species functional studies to validate mutations under clinically relevant conditions. A broader investigation of resistance mechanisms, including efflux pumps and biofilm formation, will help inform diagnostic breakpoints and antifungal stewardship.

## 5 Conclusion

This integrated study suggests that geographically associated differences in antifungal susceptibility in *D. catenulata* are linked to recurrent mutations in *ERG11* and *FKS1*, and that nutrient limitation can amplify resistance phenotypes *in vitro*. Chinese isolates showed reduced susceptibility to fluconazole and echinocandins in our collection, with clustering in Heilongjiang Province. Using heterologous expression in *S. cerevisiae*, we found that Erg11-F126L increased fluconazole MICs under SD-Ura conditions, consistent with docking results indicating altered ligand–protein interactions. Likewise, Fks1 variants conferred measurable echinocandin MIC shifts, which were more evident under SD-Ura; the combined mutant (S625L/S1123G/F1345L) produced a larger effect than individual substitutions, while Fks1-F621I and Fks1-I1348S were consistent with changes in Hotspot 1/2 regions.

Importantly, these functional results were obtained in a heterologous yeast model and under a defined nutrient-stress condition, which may not fully represent antifungal responses in *D. catenulata* during human infection. Therefore, our findings support the value of regional susceptibility monitoring and molecular surveillance of *ERG11/FKS1* hotspot mutations, while highlighting the need for future validation in the native organism and in clinically relevant models.

## Data Availability

The datasets presented in this study can be found in online repositories. The names of the repository/repositories and accession number(s) can be found in the article/**Supplementary Material**.

## References

[B1] Almeida-Paes R. Teixeira M. M. Oliveira F. A. Almeida M. A. Almeida-Silva F. Geraldo K. M. . (2024). A Cluster of Diutina catenulata Funguria in Patients with Coronavirus Disease 2019 (COVID-19) Hospitalized in a Tertiary Reference Hospital from Rio de Janeiro, Brazil. Curr. Microbiol. 81, 338. doi: 10.1007/s00284-024-03854-y, PMID: 39223407

[B2] Alsulaimany M. Keniya M. Alanazi R. N Ruma Y. Hughes C. Jones A. . (2025). Exploring long arm amide-linked side chains in the design of antifungal azole inhibitors of sterol 14α-demethylase (CYP51). J. medicinal Chem. 68, 10781–10799. doi: 10.1021/acs.jmedchem.4c02922, PMID: 40403151 PMC12169614

[B3] Arendrup M. C. Patterson T. F. (2017). Multidrug-resistant candida: epidemiology, molecular mechanisms, and treatment. J. Infect. Dis. 216, S445–s451. doi: 10.1093/infdis/jix131, PMID: 28911043

[B4] Bédard C. Gagnon-Arsenault I. Boisvert J. Plante S. Dubé A. Pageau A. . (2024). Most azole resistance mutations in the Candida albicans drug target confer cross-resistance without intrinsic fitness cost. Nat. Microbiol. 9, 3025–3040. doi: 10.1038/s41564-024-01819-2, PMID: 39379635

[B5] Berman J. Krysan D. J. (2020). Drug resistance and tolerance in fungi. Nat. Rev. Microbiol. 18, 319–331. doi: 10.1038/s41579-019-0322-2, PMID: 32047294 PMC7231573

[B6] Beyda N. D. John J. Kilic A. Alam M. J. Lasco T. M. Garey K. W. (2014). FKS mutant Candida glabrata: risk factors and outcomes in patients with candidemia. Clin. Infect. Dis. 59, 819–825. doi: 10.1093/cid/ciu407, PMID: 24879785

[B7] Çakir S. Çelebi S. Özkan H. Köksal N. Dorum B. A. Yeşil E. . (2019). Results of the use of micafungin in newborns. Mikrobiyol Bul 53, 70–80. doi: 10.5578/mb.67599, PMID: 30683041

[B8] Chen X. F. Zhang W. Fan X. Hou X. Liu X. Y. Huang J. J. . (2021). Antifungal susceptibility profiles and resistance mechanisms of clinical diutina catenulata isolates with high MIC values. Front. Cell Infect. Microbiol. 11, 739496. doi: 10.3389/fcimb.2021.739496, PMID: 34778103 PMC8586209

[B9] Díaz-García J. Gómez A. MaChado M. Alcalá L. Reigadas E. Sánchez-Carrillo C. . (2022). Blood and intra-abdominal Candida spp. from a multicentre study conducted in Madrid using EUCAST: emergence of fluconazole resistance in Candida parapsilosis, low echinocandin resistance and absence of Candida auris. J. Antimicrob. Chemother. 77, 3102–3109. doi: 10.1093/jac/dkac288, PMID: 36031723

[B10] Esfahani A. Omran A. N. Salehi Z. Shams-Ghahfarokhi M. Ghane M. Eybpoosh S. . (2022). Molecular epidemiology, antifungal susceptibility, and ERG11 gene mutation of Candida species isolated from vulvovaginal candidiasis: Comparison between recurrent and non-recurrent infections. Microb. Pathog. 170, 105696. doi: 10.1016/j.micpath.2022.105696, PMID: 35921954

[B11] Ha M. V. Choy M. S. Mccoy D. Fernandez N. Suh J. S. (2018). Candida catenulata candidaemia and possible endocarditis in a cirrhotic patient successfully de-escalated to oral fluconazole. J. Clin. Pharm. Ther. 43, 910–913. doi: 10.1111/jcpt.12728, PMID: 29956355

[B12] Hu X. Yang P. Chai C. Liu J. Sun H. Wu Y. . (2023). Structural and mechanistic insights into fungal β-1,3-glucan synthase FKS1. Nature 616, 190–198. doi: 10.1038/s41586-023-05856-5, PMID: 36949198 PMC10032269

[B13] Jospe-Kaufman M. Ben-Zeev E. Mottola A. Dukhovny A. Berman J. Carmeli S. . (2024). Reshaping echinocandin antifungal drugs to circumvent glucan synthase point-mutation-mediated resistance. Angewandte Chemie (International Ed. English) 63, e202314728. doi: 10.1002/anie.202314728, PMID: 38161189

[B14] Kekana D. Naicker S. Shuping L. Velaphi S. Nakwa F. Wadula J. . (2023). Candida auris clinical isolates associated with outbreak in neonatal unit of tertiary academic hospital, South Africa. Emerging Infect. Dis. 29, 2044–2053. doi: 10.3201/eid2910.230181, PMID: 37735719 PMC10521600

[B15] King K. González-Rodríguez L. Kaczmarek J. Jędryczka M. West J. (2025). Decreased DMI sensitivity of Plenodomus biglobosus (phoma of oilseed rape) associated with CYP51 substitution G476S. Pest Manage. science. doi: 10.1002/ps.8926, PMID: 40433890 PMC13665603

[B16] Moreau J. Noël T. Point K. Tewes F. Deroche L. Clarhaut J. . (2024). Pan-azole-resistant Meyerozyma guilliermondii clonal isolates harbouring a double F126L and L505F mutation in Erg11. Mycoses 67, e13704. doi: 10.1111/myc.13704, PMID: 38429226

[B17] Nourrisson C. Moniot M. Lavergne R. A. Robert E. Bonnin V. Hagen F. . (2023). Acquired fluconazole resistance and genetic clustering in Diutina (Candida) catenulata from clinical samples. Clin. Microbiol. Infect. 29, 257.e7–257.e11. doi: 10.1016/j.cmi.2022.09.021, PMID: 36209989

[B18] Perlin D. (2015). Echinocandin resistance in candida. Clin. Infect. diseases: an Off. Publ. Infect. Dis. Soc. America 61, S612–S617. doi: 10.1093/cid/civ791, PMID: 26567278 PMC4643482

[B19] Pristov K. E. Ghannoum M. A. (2019). Resistance of Candida to azoles and echinocandins worldwide. Clin. Microbiol. Infect. 25, 792–798. doi: 10.1016/j.cmi.2019.03.028, PMID: 30965100

[B20] Quindós G. Miranda-Cadena K. San-Millán R. Borroto-Esoda K. Cantón E. Linares-Sicilia M. J. . (2022). *In vitro* antifungal activity of ibrexafungerp (SCY-078) against contemporary blood isolates from medically relevant species of candida: A european study. Front. Cell Infect. Microbiol. 12, 906563. doi: 10.3389/fcimb.2022.906563, PMID: 35651755 PMC9149255

[B21] Radosavljevic M. Koenig H. Letscher-Bru V. Waller J. Maloisel F. Lioure B. . (1999). Candida catenulata fungemia in a cancer patient. J. Clin. Microbiol. 37, 475–477. doi: 10.1128/JCM.37.2.475-477.1999, PMID: 9889248 PMC84348

[B22] Shields R. K. Kline E. G. Healey K. R. Kordalewska M. Perlin D. S. Nguyen M. H. . (2019). Spontaneous Mutational Frequency and FKS Mutation Rates Vary by Echinocandin Agent against Candida glabrata. Antimicrob. Agents Chemother. 63, e01692-18. doi: 10.1128/AAC.01692-18, PMID: 30373796 PMC6325211

[B23] Silva S. Negri M. Henriques M. Oliveira R. Williams D. W. Azeredo J. (2012). Candida glabrata, Candida parapsilosis and Candida tropicalis: biology, epidemiology, pathogenicity and antifungal resistance. FEMS Microbiol. Rev. 36, 288–305. doi: 10.1111/j.1574-6976.2011.00278.x, PMID: 21569057

[B24] Tian Y. Zhuang Y. Chen Z. Mao Y. Zhang J. Lu R. . (2020). A gain-of-function mutation in PDR1 of Candida glabrata decreases EPA1 expression and attenuates adherence to epithelial cells through enhancing recruitment of the Mediator subunit Gal11A. Microbiol. Res. 239, 126519. doi: 10.1016/j.micres.2020.126519, PMID: 32563123

[B25] Ünal N. Spruijtenburg B. Arastehfar A. Gümral R. De Groot T. Meijer E. F. J. . (2024). Multicentre study of candida parapsilosis blood isolates in Türkiye highlights an increasing rate of fluconazole resistance and emergence of echinocandin and multidrug resistance. Mycoses 67, e70000. doi: 10.1111/myc.70000, PMID: 39547949

[B26] Williamson B. Wilk A. Guerrero K. D. Mikulski T. D. Elias T. N. Sawh I. . (2021). Impact of erg11 amino acid substitutions identified in candida auris clade III isolates on triazole drug susceptibility. Antimicrob. Agents Chemother. 66, e0162421. doi: 10.1128/AAC.01624-21, PMID: 34633842 PMC8765314

[B27] Zhang K. Tong M. Gao K. Di Y. Wang P. Zhang C. . (2015). Genomic reconstruction to improve bioethanol and ergosterol production of industrial yeast Saccharomyces cerevisiae. J. Ind. Microbiol. Biotechnol. 42, 207–218. doi: 10.1007/s10295-014-1556-7, PMID: 25475753

