# Supplementary material for: Decoding resistance in Diutina catenulata by validating clinically relevant Erg11/Fks1 mutations
Source: Front Cell Infect Microbiol. 2026 Feb 17;16:1776442. doi: 10.3389/fcimb.2026.1776442 (PMC12953444; doi:10.3389/fcimb.2026.1776442)
Supplement: Supplementary file 1 [file Table1.docx]

Supplementary Material

# Supplementary Data

**(A)**

Target gene：*ERG11*

Antifungal drug: Fluconazole

1. *D. catenulata Strain* 15H1506 (wild type)

Gene sequence（1563bp）

ATGGCGCTCGCAGACTACGTTGTGCTGGCGACTACGGCGTTTCTTGCTCTCAGTACTGCCCAGCAGTTGCTGATTCTTGTGGGTGTTCCCTTCATCTACCACCTTTTATGGCAGTTCGTCTACCTGTTGCGTAAGGACCGTGCGCCGTTAGTGTTCCACTGGATCCCCTGGGTGGGGCTGGCGGTCACCTACGGCATGAGACCGTACGAGTTTTTTGAGGAGTGCCGTCTCAAGTACGGCGATGTGTTTGCCTTTGTGTTGATGGGCCGGGTGATGACGGTGTACCTTGGTCCGAAGGGCCACGAATTTGTCTTCAACCTGAAGTTGGCCGATGTGTCCGCTGAGGAGGCATACAAGCACTTGACTACCCCTGTGTTCGGTAAGGGTGTCGTCTATGACTGCCCCAACCACCGGTTGATGGAGCAAAAGAAGTTTGCCAAGTTTGCCTTGACCACCGACCTGTTCCGCCAGTACGTGCCTCAGATCCGCGAAGAGATTTTGAAGTACTTTGACACCGGTTTCCACATGAAGACAAAGAAGTCTGACTCGGTCAATGTCATGAAGACCCAGCCCGAGGTGACGATTTTCACTGCCCTGCGCTCGTTGCTCGGCGAGCAGATGCGTGAGAAGTTGGACGAGCTGTTTGCTCAGTTGTACCTGGACTTGGACAAGGGGTTCACCCCCATCAACTTTGTGTTCCCCAACCTCCCGTTGCCTCTTTACTGGAAGCGTGACAACGCCCAGCGCAAGATTTCGGGTACGTACATGGATTTGATTGCCACTCGCCGGTCCACCGGGGACATTGACCCCAAGCGCGACCTTGTTGACTCGTTGATGGTCAACCTGACCTATAAGGACGGTGTCAAGATGACCGACCAGGAGATTGCCAACTTGTTAATTGGCGTGCTCATGGGTGGCCAGCACACCCTGGCGGCCACCTCTGCTTGGTTTTTGCTCCACTTGGGTGAGAAGCCCGAGTTGCAGGAGGAGTTGTACCACGAGGTCAAGCACGTGCTTGACCAGAAGGGTGGCAACTTGAACGACTTGTCATACGAGGACTTGCAGTCAATGCCCTTGGTTAACAACGTCATTAAAGAGACTTTGCGTATGCACTCGCCTTTGCACTCGATTTTCCGTAAGGTGATGCGTCCATTGGTGGTTCCCGGCACCCAGTACGTGGTTCCCAAGGGCCACCACGTGCTTGTTTCGCCTGGTTACACCATGACTCTGGAGCGATTCTTCCCTCACGCGGCTGAGTTTGACCCTCACCGGTGGGACGAGGTTAAGGCGGAGGAGGCGCAGGAGCTGGTTGACTACGGGTTTGGTGCTGTGAACAAGGGTGTATCGCTGCCTTACCTTCCCTTCGGTGGTGGTCGCCACCGTTGTATCGGTGAGCAGTTTGCCTACGTTCAGTTGGGCACGATCTTGGCTACCTATGTGTACAACTTGAAGTGGACTTTGGACGGGAAGTTGCCACTCCCCGATTTCCAATCGATGGTGACTTTGCCTTTGATTGAGGACGCCAACATTACTTGGGAAAAGCGGGATACTTGCGTGTTATAG

Amino Acid Sequence（520aa）

MALADYVVLATTAFLALSTAQQLLILVGVPFIYHLLWQFVYLLRKDRAPLVFHWIPWVGLAVTYGMRPYEFFEECRLKYGDVFAFVLMGRVMTVYLGPKGHEFVFNLKLADVSAEEAYKHLTTPVFGKGVVYDCPNHRLMEQKKFAKFALTTDLFRQYVPQIREEILKYFDTGFHMKTKKSDSVNVMKTQPEVTIFTALRSLLGEQMREKLDELFAQLYLDLDKGFTPINFVFPNLPLPLYWKRDNAQRKISGTYMDLIATRRSTGDIDPKRDLVDSLMVNLTYKDGVKMTDQEIANLLIGVLMGGQHTLAATSAWFLLHLGEKPELQEELYHEVKHVLDQKGGNLNDLSYEDLQSMPLVNNVIKETLRMHSPLHSIFRKVMRPLVVPGTQYVVPKGHHVLVSPGYTMTLERFFPHAAEFDPHRWDEVKAEEAQELVDYGFGAVNKGVSLPYLPFGGGRHRCIGEQFAYVQLGTILATYVYNLKWTLDGKLPLPDFQSMVTLPLIEDANITWEKRDTCVL*

2.*D. catenulata* Strain 16HLJ6019 (C378G[F126L] mutation)

Gene sequence（1563bp）

ATGGCGCTCGCAGACTACGTTGTGCTGGCGACTACGGCGTTTCTTGCTCTCAGTACTGCCCAGCAGTTGCTGATTCTTGTGGGTGTTCCCTTCATCTACCACCTTTTATGGCAGTTCGTCTACCTGTTGCGTAAGGACCGTGCGCCGTTAGTGTTCCACTGGATCCCCTGGGTGGGGCTGGCGGTCACCTACGGCATGAGACCGTACGAGTTTTTTGAGGAGTGCCGTCTCAAGTACGGCGATGTGTTTGCCTTTGTGTTGATGGGCCGGGTGATGACGGTGTACCTTGGTCCGAAGGGCCACGAGTTTGTCTTCAACCTGAAGTTGGCCGATGTGTCCGCTGAGGAGGCATACAAGCACTTGACTACCCCTGTGTTGGGTAAGGGTGTCGTCTATGACTGCCCCAACCACCGGTTKATGGAGCAAAAGAAGTTTGCCAAGTTTGCCTTGACCACCGACCTGTTCCGCCAGTACGTGCCTCAGATCCGCGAAGAGATTTTGAAGTACTTTGACACCGGTTTCCACATGAAGACAAAGAAGTCTGACTCGGTCAATGTCATGAAGACCCAGCCCGAGGTGACGATTTTCACTGCCCTGCGCTCGTTGCTCGGCGAGCAGATGCGTGAGAAGTTGGACGAGCTGTTTGCTCAGTTGTACCTGGACTTGGACAAGGGGTTCACTCCCATCAACTTTGTGTTCCCCAACCTCCCGTTGCCTCTTTACTGGAAGCGTGACAACGCCCAGCGCAAGATTTCGGGTACGTACATGGATTTGATTGCCACTCGCCGGTCCACCGGGGACATTGACCCCAAGCGCGACCTTGTTGACTCGTTGATGGTCAACCTGACCTACAAGGACGGTGTCAAGATGACCGACCAGGAGATTGCAAACTTGTTAATTGGCGTGCTCATGGGTGGCCAGCACACCCTGGCGGCCACCTCTGCTTGGTTTTTGCTCCACTTGGGTGAGAAGCCCGAGTTGCAGGAGGAGTTGTACCATGAGGTCAAGCACGTGCTTGACCAGAAGGGTGGCAACTTGAACGACTTGTCATACGAGGACTTGCAGTCAATGCCCTTGGTTAACAACGTCATTAAAGAGACTTTGCGTATGCACTCGCCTTTGCACTCGATTTTCCGTAAGGTGATGCGTCCATTGGTGGTTCCTGGCACCCAGTACGTGGTTCCCAAGGGCCACCACGTGCTTGTTTCGCCTGGTTACACCATGACTCTGGAGCGATTCTTCCCTCACGCGGCTGAGTTTGACCCTCACCGGTGGGACGAGGTTAAGGCGGAGGAGGCGCAGGAGCTGGTTGACTACGGGTTTGGTGCTGTGAACAAGGGTGTATCGCTGCCTTACCTTCCCTTCGGTGGTGGTCGCCACCGTTGTATCGGTGAGCAGTTTGCCTACGTTCAGTTGGGCACGATCTTGGCTACCTATGTGTACAACTTGAAGTGGACTTTGGACGGGAAGTTGCCACTCCCCGATTTCCAATCGATGGTGACTTTGCCTTTGATTGAGGACGCCAACATTACTTGGGAAAAGCGGGATACTTGCGTGTTATAG

Amino Acid Sequence（520aa）

MALADYVVLATTAFLALSTAQQLLILVGVPFIYHLLWQFVYLLRKDRAPLVFHWIPWVGLAVTYGMRPYEFFEECRLKYGDVFAFVLMGRVMTVYLGPKGHEFVFNLKLADVSAEEAYKHLTTPVLGKGVVYDCPNHRFMEQKKFAKFALTTDLFRQYVPQIREEILKYFDTGFHMKTKKSDSVNVMKTQPEVTIFTALRSLLGEQMREKLDELFAQLYLDLDKGFTPINFVFPNLPLPLYWKRDNAQRKISGTYMDLIATRRSTGDIDPKRDLVDSLMVNLTYKDGVKMTDQEIANLLIGVLMGGQHTLAATSAWFLLHLGEKPELQEELYHEVKHVLDQKGGNLNDLSYEDLQSMPLVNNVIKETLRMHSPLHSIFRKVMRPLVVPGTQYVVPKGHHVLVSPGYTMTLERFFPHAAEFDPHRWDEVKAEEAQELVDYGFGAVNKGVSLPYLPFGGGRHRCIGEQFAYVQLGTILATYVYNLKWTLDGKLPLPDFQSMVTLPLIEDANITWEKRDTCVL*

3.*D. catenulata* Strain 10H1051 (A428G[K143R] mutation)

Gene sequence（1563bp）

ATGGCGCTCGCAGACTACGTTGTGCTGGCGACTACGGCGTTTCTTGCTCTCAGTACTGCCCAGCAGTTGCTGATTCTTGTGGGTGTTCCCTTCATCTACCACCTTTTATGGCAGTTCGTCTACCTGTTGCGTAAGGACCGTGCGCCGTTAGTGTTCCACTGGATCCCCTGGGTGGGGCTGGCGGTCACCTACGGCATGAGACCGTACGAGTTTTTTGAGGAGTGCCGTCTCAAGTACGGCGATGTGTTTGCCTTTGTGTTGATGGGCCGGGTGATGACGGTGTACCTTGGTCCGAAGGGCCACGAGTTTGTCTTCAACCTGAAGTTGGCCGATGTGTCCGCTGAGGAGGCATACAAGCACTTGACTACCCCTGTGTTCGGTAAGGGTGTCGTCTATGACTGCCCCAACCACCGGTTGATGGAGCAAAGGAAGTTTGCCAAGTTTGCCTTGACCACCGACCTGTTCCGCCAGTACGTGCCTCAGATCCGCGAAGAGATTTTGAAGTACTTTGACACCGGTTTCCACATGAAGACAAAGAAGTCTGACTCGGTCAATGTCATGAAGACCCAGCCCGAGGTGACGATTTTCACTGCCCTGCGCTCGTTGCTCGGCGAGCAGATGCGTGAGAAGTTGGACGAGCTGTTTGCTCAGTTGTACCTGGACTTGGACAAGGGGTTCACTCCCATCAACTTTGTGTTCCCCAACCTCCCGTTGCCTCTTTACTGGAAGCGTGACAACGCCCAGCGCAAGATTTCGGGTACGTACATGGATTTGATTGCCACTCGCCGGTCCACCGGGGACATTGACCCCAAGCGCGACCTTGTTGACTCGTTGATGGTCAACCTGACCTACAAGGACGGTGTCAAGATGACCGACCAGGAGATTGCAAACTTGTTAATTGGCGTGCTCATGGGTGGCCAGCACACCCTGGCGGCCACCTCTGCTTGGTTTTTGCTCCACTTGGGTGAGAAGCCCGAGTTGCAGGAGGAGTTGTACCATGAGGTCAAGCACGTGCTTGACCAGAAGGGTGGCAACTTGAACGACTTGTCATACGAGGACTTGCAGTCAATGCCCTTGGTTAACAACGTCATTAAAGAGACTTTGCGTATGCACTCGCCTTTGCACTCGATTTTCCGTAAGGTGATGCGTCCATTGGTGGTTCCTGGCACCCAGTACGTGGTTCCCAAGGGCCACCACGTGCTTGTTTCGCCTGGTTACACCATGACTCTGGAGCGATTCTTCCCTCACGCGGCTGAGTTTGACCCTCACCGGTGGGACGAGGTTAAGGCGGAGGAGGCGCAGGAGCTGGTTGACTACGGGTTTGGTGCTGTGAACAAGGGTGTATCGCTGCCTTACCTTCCCTTCGGTGGTGGTCGCCACCGTTGTATCGGTGAGCAGTTTGCCTACGTTCAGTTGGGCACGATCTTGGCTACCTATGTGTACAACTTGAAGTGGACTTTGGACGGGAAGTTGCCACTCCCCGATTTCCAATCGATGGTGACTTTGCCTTTGATTGAGGACGCCAACATTACTTGGGAAAAGCGGGATACTTGCGTGTTATAG

Amino Acid Sequence（520aa）

MALADYVVLATTAFLALSTAQQLLILVGVPFIYHLLWQFVYLLRKDRAPLVFHWIPWVGLAVTYGMRPYEFFEECRLKYGDVFAFVLMGRVMTVYLGPKGHEFVFNLKLADVSAEEAYKHLTTPVFGKGVVYDCPNHRLMEQRKFAKFALTTDLFRQYVPQIREEILKYFDTGFHMKTKKSDSVNVMKTQPEVTIFTALRSLLGEQMREKLDELFAQLYLDLDKGFTPINFVFPNLPLPLYWKRDNAQRKISGTYMDLIATRRSTGDIDPKRDLVDSLMVNLTYKDGVKMTDQEIANLLIGVLMGGQHTLAATSAWFLLHLGEKPELQEELYHEVKHVLDQKGGNLNDLSYEDLQSMPLVNNVIKETLRMHSPLHSIFRKVMRPLVVPGTQYVVPKGHHVLVSPGYTMTLERFFPHAAEFDPHRWDEVKAEEAQELVDYGFGAVNKGVSLPYLPFGGGRHRCIGEQFAYVQLGTILATYVYNLKWTLDGKLPLPDFQSMVTLPLIEDANITWEKRDTCVL*

**（B）**

Target gene : FKS1 gene

Antifungal drug : Caspofungin

1.*D. catenulata* Strain 15H1506 (wild type)

Gene sequence（5652bp）

ATGTCGTACAACGATAACCATCATAATTATTATGACCCAAATGCCCCCGAGGGGGCAGATGGGTACTACCAGGCCCCGTATGACGGTGGTCAGGGCCCCCAGGAGTACTACGACCCCAACATGCAGTACCAGCAAGGGGCGCCCGAGATGGAAGGTGGCTACGACCAGGGTTACCACCAGCAGGGCCAGTACGGTGGTGAGGCGTTCCTGGATTTCAGCTACGGCGGCCAGGGCGGGTACGACCAGTACAACGGCACCCAGTACACCCCGCTGCAGATGAGTTACGGTGGGGACCCGCGGTCGCTGGGGGCGCTGACGCCAATCTACGGTGGCCAGGGCCAGTACGACCCTACCCAGTTCCAGATGCTGCTGAACTTGCCGTACCCGGCGTGGCTGGCTGACCCCCAGGCCCCGATCAAGGTGGAGCACATTGAGGACATTTTCATTGACCTTGCCAACAAATTTGGGTTCCAGCGGGACCTGATGCGTAACATGTTCGACTACTTCATGACGTTGTTGGACCTGCGGTCGCTGCGGATGCTGCCGGCGCAGGCGCTTTTGCTGTTGCACGCCGACTACATTGGTGGTGAGAACGCCAACTACCGCAAGTGGTACTTCGCTTCGCAGCAGGACTTGGACGACTCGGTGGGCTACGCCAACATGCAGTTGGGTAAGATTGGTCGCAAGGCCCGCAAGGCTAAGCGGAAGCTGAAGAAGGCTCGTCAGGCTGCCGAAGAGATTGGCCAAGACGTTGACGCTCTCGACAACGAGTTGGAGGGCGACTACTCTTTGGAGGCGGCCGAGATCCGCTGGAAGGCCAAGATGAATGCGTTGCTGCCCGAGGAGCGTGTTCGTGACATTGCCCTTTACTTATTGATGTGGGGTGAGGCCAACCAGATCCGTTTTACTCCCGAGTGCTTGTGTTTCTTGTTCAAGCTGGCGATGGACTACTTGATGTCGCCCGCGTGCCAGCAGCGTACCGAGCCTGTGCCTGAGGGTGACTACCTTAACCGGGTCATCACTCCTCTTTACCGGTTTTTGCGGTCGCAGGTGTACGAAATCTACGAGGGCCGGTTCGTCAAGCGTGAGAAGGACCACAACAAGGTGATTGGTTACGACGACGTTAACCAGTTGTTCTGGTACCCCGAGGGTATCCTGCGTATCATTTTTGAGGATGGTACCCGACTCGTCGATATGCCTCAAGCCGAGCGGTGGGGCCGCTTGGGTGAGGTTGAGTGGAACAACGTTTTCTTCAAGACCTACAAGGAGATTCGTACCTGGTTGCATTTCGTCACCAACTTTAACCGTATTTGGATTATCCACGGTACCATCTACTGGATGTACACGGCCTACAACTCTCCCACTTTGTACACCCAGAACTATGTGCAGACCATGAACCAGCAGCCGTTGGCGCTGTCTCGTTGGGCATCTGCCGCTATCGGTGGTATTCTTGCCTCCGCCATTCAGATTTTTGCTACCCTCTTCGAGTGGATGTTCGTTCCTCGTGAATGGGCGGGTGCTCAGCACCTTTCGCGCCGGTTGGGTTTCCTTGTGTTGATTTTGTTGTTGAACTTGGTGCCCGTTGTTTACACTTTCTACTGGGCCGGTTTGCTGTCTTACTCGAAGCTGGCGCACGCTGTGTCGATTGTCGGTTTCTTCATTGCCATCGCTACCCTTTTGTTCTACGCGGTTATGCCTTTGGGTGGCTTGTTCACCCTGTACATGAACAAGTCGCTGCGTAAGTACTTGGCGTCGCAGACTTTCACTGCCAACTTTATCAAGTTGCGTGGCATTGACATGTGGATGTCCTACCTTTTGTGGGTGCTCGTTTTCCTTGCCAAGCTCGTTGAATCGTACTTCTTCCTTACCCTTTCGTTGCGTGACCCTGTTCGTAACTTGCTGACGATGACGATGCGGTGCATTGGTGAGCTGTGGTGGGGGTTCACCCTCTGTCGCCAGCAGGCCAAGGTTGTGTTGGGTTTGATGTACGCCGTCGACCTTTTGTTGTTCTTCCTTGATACCTACATGTGGTACATTATCTGTAACTGTATCTTCTCGATTGGTCGCTCGTTCTACCTCGGTATCTCCATTTTGACTCCTTGGCGTAACATCTTCACTCGGTTGCCCAAGCGTATCTACTCTAAGATCCTTGCCACCACCGAGATGGAGATCAAGTACAAGCCCAAGGTGTTGATCTCGCAGATTTGGAACGCTATTGTCATCTCCATGTACCGTGAGCACTTGTTGGCCATTGACCACGTTCAGAAGTTGTTGTACCACCAGGTGCCGTCCGAGATTGAGGGCAAGCGCACTCTCCGTGCACCCACTTTCTTCGTGCTGCAGGACGACAACAACTTTGAGACCGAGTTCTTCCCCCGTAACTCTGAAGCCGAGCGTCGTATTCTGTTCTTTGCTCAGTCGTTGGCTACCCCCATCTTGGAGCCTTTGCCCGTGGACAACATGCCCACTTTCACTGTGTTTACTCCTCATTACTCTGAAAAGATTTTGTTGTCTTTGCGGGAGATTATTCGTGAGGATGACCAATACTCGCGGGTGACCCTTTTGGAGTACTTGAAGCAGTTGCACCCTGTCGAGTGGGACTGTTTCGTTAAGGACACCAAGATTTTGGCTGAGGAGACTGCTGCTTACGAGAACGGTGACGAGGAGAAGGCTTCCGAAGATGACGGACTCAAGTCCAAGATCGACGATTTGCCCTTCTACTGCATTGGTTTCAAGCTGGCTGCCCCGGAGTACACTCTTCGTACTCGTATTTGGGCTTCGTTGCGTTCGCAGACTTTGTACCGTACTGTTTCTGGTTTCATGAACTACGCCCGTGCCATTAAGTTGTTGTACCGGGTGGAGAACCCTGAACTTGTCCAGTACTTTGGTGGTGACCCCGAGGGTCTCGAATTGGCTTTGGAAAAGATGGCTCGTCGTAAGTTCCGCTTCCTTGTGCTGATGCAGCGTATGGCTAAGTTTAAGGACGACGAGATGGAGAACGCTGAGTTCTTGTTGCGTGCCTACCCCGACTTGCAGATTGCCTACCTCGACGAGGAGCCGGCGATGAACGAGGAAGAGGAGCCGCGGGTGTACTCGGCTTTGATTGATGGTCACTGCGAGATGCTCGAAAACGGCCGTCGTCGTCCTAAGTTCCGGGTCCAGTTGTCGGGTAACCCCATTCTCGGTGACGGTAAGTCTGATAACCAGAACCATGCCATTATTTTCCACCGTGGTGAGTACATCCAGTTGATTGACGCTAACCAGGACAACTACTTGGAGGAGTGTCTTAAGATTCGTTCGGTTTTGGCTGAATTCGAGGAGCTCAACGTTGAGCACGTCAACCCTTACGCCCCTGAGCTTAAGGACAGCACTCCCAAGGAGAAGCGCTACCCTGTTGCCTTCCTTGGTGCTCGTGAATACATTTTCTCGGAGAACTCTGGTGTTCTTGGTGACGTTGCTGCTGGTAAGGAACAGACTTTCGGTACTTTGTTTGCTCGTACTTTGGCTCAGATTGGCGGTAAGTTGCACTACGGTCACCCCGATTTCTTGAACGCCACGTTCATGTTGACTCGTGGTGGTGTGTCCAAGGCCCAGAAGGGTTTGCATCTTAACGAAGATATTTATGCCGGTATGAACGCCATGTTGCGTGGTGGTAAGATTAAGCACTGCGAGTACTACCAGTGTGGTAAGGGTCGTGATATGGGTTTCGGTTCCATTTTGAACTTTACCACCAAGATTGGTGCTGGTATGGGTGAACAGATGTTGTCGCGTGAGTACTACTACCTTTCTACGCAGTTGCCCCTTGACCGTTTCCTTTCGTTCTACTACGGTCACCCTGGTTTCCACATTAACAACTTGTTCATTCAGATGTCTTTGCAGGTCTTCATTTTGGTGTTGGCTAACCTTAACTCGTTGGCCCACGAGCTGATTATTTGTTCTTACAACAAGGACTTGCCCGTGACCGATTTGTTGTACCCCCTTGGCTGCTACAACTTGGTTCCCGCTGTTGACTGGATTCGTCGTTACACCCTTTCCATTTTCATTGTGTTCTTCATTTCGTTTATTCCCTTGACGGTGCAAGAATTGATTGAGCGTGGTGTGTGGAAGGCGTTCCAGCGTTTCGTCCGTCACTTGATCTCGTTGTCGCCCATGTTTGAGGTTTTCGTTGCTCAGATTTACTCGTCTTCGATTGCTACCGACATGACTGTGGGTGGTGCCCGGTATATCTCGACTGGTCGTGGTTTCGCTACCCTGCGTATCCCCTTCTCGATTTTGTACCTGCGGTTCGCTGACCTGTCTATTTACATGGGTGCTCGTCTTATGTTGATTTTGTTCTTTGGTTCCGTGGCTCACTGGCAGGCTCCCTTGTTGTGGTTCTGGGCCTGTTTGACTGCGTTGATCATGTCTCCTTTCATTTTCAACCCCCACCAGTTTGCTTGGGAGGACTTCTTCATTGACTACCGTGACTTCATTCGCTGGTTGTCTCGTGGTAACACAAAGTGGCACCGCAACTCTTGGATTGGCTACGTGCGTCTCTCGCGGTCGCGTGTCACTGGTTTCAAGCGCAAGTTGCTCAACGATGTTTCTGAGAAGCTGGCTGGTGATGCTCTGCGTGCTCACCGTTCTAACGTCTTCATGGCGTCGATCTTGCCCACTGCCGTCTACACCGCTGGTTTGTTTGTTGCCTACACTTTCATCAACGCCCAGACTGGTGTCACCGAGTACGTGGACAAGGACGGTAACACTGCTCCTGCTGGTCCCGTCAACTCGGTCTTGCGTCTTATTATCTGTGCGCTTGGTCCGGTTGTGATCAACGCTGGTGTTGTCGGTGGCTGTTTTGGTTTGGCTTGTTGCGCTGGTCCCATGTTGGGCATGTGCTGCAAGAAGACCGGTGCCGTCATGGCTGGTATTGCTCACGGTGTTGCTGTTGTCATTCACATTGTGTTCTTCATTGTGATGTGGGTGATGGAGGGCTTTAACTTTGCTCGTATGTTGCTTGGTTTGGCCACCTGTATTTACGTGCAGCGTCTCTTGTTCAAGGTTCTTACTGTGTTGTGTCTTACCCGTGAGTTCAAGAACGACCGTGCTAACACGGCTTTCTGGACCGGTAAGTGGTACAACACCGGTATGGGCTGGATGGCGGTGACCCAGCCTGCTCGTGAATACGTTGCTAAGATTGTGGAGATGTCGGAGTTCGCTGGTGACTTTATGTTGGCTCACCTCATCTTGTTTGTCCAGTTGCCCATCTTGTTCATTCCCCTCATTGACAGATGGCACCTGACCATGTTGTTCTGGTTGAAGCCCCTGCAGTTGATCCGTCCTCCCATCTACTCGCTTAAGCAGGCCCGCTTGCGGAAGCGTATGGTGCGCAAGTACTGTACTTTATACTTCGCTGTCCTCGTGTTGTTCATCGTCATTATTGTCGCCCCCGCGGTGGCCCTGTCTCACATTGACAACATCGGTGACAAGTTGGAGAACATCGATGATGGTGGCATTGGGTTGTCTGGCTTGTTCCAGCCCCGTAACACTCTGAACAACGACACTGGCCCCAACATGCCCTCGGGTGGCCTGTGGTTGTCGCTGGCTTGGTCTTTCAATTCGATCAAGTTCACCGGCACCCTTTCGTCGTACACTACCAAGGAGTTCAAGTGA

Amino Acid Sequence（1883aa）

MSYNDNHHNYYDPNAPEGADGYYQAPYDGGQGPQEYYDPNMQYQQGAPEMEGGYDQGYHQQGQYGGEAFLDFSYGGQGGYDQYNGTQYTPLQMSYGGDPRSLGALTPIYGGQGQYDPTQFQMLLNLPYPAWLADPQAPIKVEHIEDIFIDLANKFGFQRDLMRNMFDYFMTLLDLRSLRMLPAQALLLLHADYIGGENANYRKWYFASQQDLDDSVGYANMQLGKIGRKARKAKRKLKKARQAAEEIGQDVDALDNELEGDYSLEAAEIRWKAKMNALLPEERVRDIALYLLMWGEANQIRFTPECLCFLFKLAMDYLMSPACQQRTEPVPEGDYLNRVITPLYRFLRSQVYEIYEGRFVKREKDHNKVIGYDDVNQLFWYPEGILRIIFEDGTRLVDMPQAERWGRLGEVEWNNVFFKTYKEIRTWLHFVTNFNRIWIIHGTIYWMYTAYNSPTLYTQNYVQTMNQQPLALSRWASAAIGGILASAIQIFATLFEWMFVPREWAGAQHLSRRLGFLVLILLLNLVPVVYTFYWAGLLSYSKLAHAVSIVGFFIAIATLLFYAVMPLGGLFTLYMNKSLRKYLASQTFTANFIKLRGIDMWMSYLLWVLVFLAKLVESYFFLTLSLRDPVRNLLTMTMRCIGELWWGFTLCRQQAKVVLGLMYAVDLLLFFLDTYMWYIICNCIFSIGRSFYLGISILTPWRNIFTRLPKRIYSKILATTEMEIKYKPKVLISQIWNAIVISMYREHLLAIDHVQKLLYHQVPSEIEGKRTLRAPTFFVLQDDNNFETEFFPRNSEAERRILFFAQSLATPILEPLPVDNMPTFTVFTPHYSEKILLSLREIIREDDQYSRVTLLEYLKQLHPVEWDCFVKDTKILAEETAAYENGDEEKASEDDGLKSKIDDLPFYCIGFKLAAPEYTLRTRIWASLRSQTLYRTVSGFMNYARAIKLLYRVENPELVQYFGGDPEGLELALEKMARRKFRFLVLMQRMAKFKDDEMENAEFLLRAYPDLQIAYLDEEPAMNEEEEPRVYSALIDGHCEMLENGRRRPKFRVQLSGNPILGDGKSDNQNHAIIFHRGEYIQLIDANQDNYLEECLKIRSVLAEFEELNVEHVNPYAPELKDSTPKEKRYPVAFLGAREYIFSENSGVLGDVAAGKEQTFGTLFARTLAQIGGKLHYGHPDFLNATFMLTRGGVSKAQKGLHLNEDIYAGMNAMLRGGKIKHCEYYQCGKGRDMGFGSILNFTTKIGAGMGEQMLSREYYYLSTQLPLDRFLSFYYGHPGFHINNLFIQMSLQVFILVLANLNSLAHELIICSYNKDLPVTDLLYPLGCYNLVPAVDWIRRYTLSIFIVFFISFIPLTVQELIERGVWKAFQRFVRHLISLSPMFEVFVAQIYSSSIATDMTVGGARYISTGRGFATLRIPFSILYLRFADLSIYMGARLMLILFFGSVAHWQAPLLWFWACLTALIMSPFIFNPHQFAWEDFFIDYRDFIRWLSRGNTKWHRNSWIGYVRLSRSRVTGFKRKLLNDVSEKLAGDALRAHRSNVFMASILPTAVYTAGLFVAYTFINAQTGVTEYVDKDGNTAPAGPVNSVLRLIICALGPVVINAGVVGGCFGLACCAGPMLGMCCKKTGAVMAGIAHGVAVVIHIVFFIVMWVMEGFNFARMLLGLATCIYVQRLLFKVLTVLCLTREFKNDRANTAFWTGKWYNTGMGWMAVTQPAREYVAKIVEMSEFAGDFMLAHLILFVQLPILFIPLIDRWHLTMLFWLKPLQLIRPPIYSLKQARLRKRMVRKYCTLYFAVLVLFIVIIVAPAVALSHIDNIGDKLENIDDGGIGLSGLFQPRNTLNNDTGPNMPSGGLWLSLAWSFNSIKFTGTLSSYTTKEFK*

2.*D. catenulata* Strain 13TJ359 (A3367G[S1123G] mutation)

Gene sequence（5652bp）

ATGTCGTACAACGATAACCATCATAATTATTATGACCCAAATGCCCCCGAGGGGGCAGATGGGTACTACCAGGCCCCGTATGACGGTGGTCAGGGCCCCCAGGAGTACTACGACCCCAACATGCAGTACCAGCAAGGGGCGCCCGAGATGGAAGGTGGCTACGACCAGGGTTACCACCAGCAGGGCCAGTACGGTGGTGAGGCGTTCCTGGATTTCAGCTACGGCGGCCAGGGCGGGTACGACCAGTACAACGGCACCCAGTACACCCCGCTGCAGATGAGTTACGGTGGGGACCCGCGGTCGCTGGGGGCGCTGACGCCAATCTACGGTGGCCAGGGCCAGTACGACCCTACCCAGTTCCAGATGCTGCTGAACTTGCCGTACCCGGCGTGGCTGGCTGACCCCCAGGCCCCGATCAAGGTGGAGCACATTGAGGACATTTTCATTGACCTTGCCAACAAATTTGGGTTCCAGCGGGACCTGATGCGTAACATGTTCGACTACTTCATGACGTTGTTGGACCTGCGGTCGCTGCGGATGCTGCCGGCGCAGGCGCTTTTGCTGTTGCACGCCGACTACATTGGTGGTGAGAACGCCAACTACCGCAAGTGGTACTTCGCTTCGCAGCAGGACTTGGACGACTCGGTGGGCTACGCCAACATGCAGTTGGGTAAGATTGGTCGCAAGGCCCGCAAGGCTAAGCGGAAGCTGAAGAAGGCTCGTCAGGCTGCCGAAGAGATTGGCCAAGACGTTGACGCTCTCGACAACGAGTTGGAGGGCGACTACTCTTTGGAGGCGGCCGAGATCCGCTGGAAGGCCAAGATGAATGCGTTGCTGCCCGAGGAGCGTGTTCGTGACATTGCCCTTTACTTATTGATGTGGGGTGAGGCCAACCAGATCCGTTTTACTCCCGAGTGCTTGTGTTTCTTGTTCAAGCTGGCGATGGACTACTTGATGTCGCCCGCGTGCCAGCAGCGTACCGAGCCTGTGCCTGAGGGTGACTACCTTAACCGGGTCATCACTCCTCTTTACCGGTTTTTGCGGTCGCAGGTGTACGAAATCTACGAGGGCCGGTTCGTCAAGCGTGAGAAGGACCACAACAAGGTGATTGGTTACGACGACGTTAACCAGTTGTTCTGGTACCCCGAGGGTATCCTGCGTATCATTTTTGAGGATGGTACCCGACTCGTCGACATGCCTCAGGCCGAGCGGTGGGGCCGCTTGGGTGAGGTTGAGTGGAACAACGTTTTCTTCAAGACCTACAAGGAGATTCGTACCTGGTTGCATTTCGTCACCAACTTTAACCGTATTTGGATTATCCACGGTACCATCTACTGGATGTACACGGCCTACAACTCTCCCACTTTGTACACCCAGAACTATGTGCAGACCATGAACCAGCAGCCGTTGGCGCTGTCTCGTTGGGCATCTGCCGCTATCGGTGGTATTCTTGCCTCCGCCATTCAGATTTTTGCTACCCTCTTCGAGTGGATGTTCGTTCCTCGTGAATGGGCGGGTGCTCAGCACCTTTCGCGCCGGTTGGGTTTCCTTGTATTGATTTTGTTGTTGAACTTGGTGCCCGTTGTTTACACTTTCTACTGGGCCGGTTTGCTGTCTTACTCGAAGCTGGCGCACGCTGTGTCGATTGTCGGTTTCTTCATTGCCATCGCTACCCTTTTGTTCTACGCGGTTATGCCTTTGGGTGGCTTGTTCACCCTGTACATGAACAAGTCGCTGCGTAAGTACTTGGCGTCGCAGACTTTCACTGCCAACTTTATCAAGTTGCGTGGCATTGACATGTGGATGTCCTACCTTTTGTGGGTGCTCGTTTTCCTTGCCAAGCTCGTTGAATCGTACTTCTTCCTTACCCTTTCGTTGCGTGACCCTGTTCGTAACTTGCTGACGATGACGATGCGGTGCATTGGTGAGCTGTGGTGGGGGTTCACCCTCTGTCGCCAGCAGGCCAAGGTTGTGTTGGGTTTGATGTACGCCGTCGACCTTTTGTTGTTCTTCCTTGATACCTACATGTGGTACATTATCTGTAACTGTATCTTCTCGATTGGTCGCTCGTTCTACCTCGGTATCTCCATTTTGACTCCCTGGCGTAACATCTTCACTCGGTTGCCCAAGCGTATCTACTCTAAGATCCTTGCCACCACCGAGATGGAGATCAAGTACAAGCCCAAGGTGTTGATCTCGCAGATTTGGAACGCTATTGTCATCTCCATGTACCGTGAGCACTTGTTGGCCATTGACCACGTTCAGAAGTTGTTGTACCACCAGGTGCCGTCCGAGATTGAGGGCAAGCGCACTCTCCGTGCACCCACTTTCTTCGTGCTGCAGGACGACAACAACTTTGAGACCGAGTTCTTCCCCCGTAACTCTGAAGCCGAGCGTCGTATTCTGTTCTTTGCTCAGTCGTTGGCTACCCCCATCTTGGAGCCTTTGCCCGTGGACAACATGCCCACTTTCACTGTGTTTACTCCTCATTACTCTGAAAAGATTTTGTTGTCTTTGCGGGAGATTATTCGTGAGGATGACCAATACTCGCGGGTGACCCTTTTGGAGTACTTGAAGCAGTTGCACCCTGTCGAGTGGGACTGTTTCGTTAAGGACACCAAGATTTTGGCTGAGGAGACTGCTGCTTACGAGAACGGTGACGAGGAGAAGGCTTCCGAAGATGACGGACTCAAGTCCAAGATCGACGATTTGCCCTTCTACTGCATTGGTTTCAAGCTGGCTGCCCCGGAGTACACTCTTCGTACTCGTATTTGGGCTTCCTTGCGTTCGCAGACTTTGTACCGTACTGTTTCTGGTTTCATGAACTACGCCCGTGCCATTAAGTTGTTGTACCGGGTGGAGAACCCTGAGCTTGTCCAGTACTTTGGTGGTGACCCCGAGGGTCTCGAATTGGCTTTGGAAAAGATGGCTCGTCGTAAGTTCCGCTTCCTTGTGCTGATGCAGCGTATGGCTAAGTTTAAGGACGACGAGATGGAGAACGCTGAGTTCTTGTTGCGTGCCTACCCCGACTTGCAAATTGCCTACCTCGACGAGGAGCCGGCGATGAACGAGGAAGAGGAGCCGCGGGTGTACTCGGCTTTGATTGATGGTCACTGCGAGATGCTCGAAAACGGCCGTCGTCGTCCTAAGTTCCGGGTCCAGTTGTCGGGTAACCCCATTCTCGGTGACGGTAAGTCTGATAACCAGAACCATGCCATTATTTTCCACCGTGGTGAGTACATCCAGTTGATTGACGCTAACCAGGACAACTACTTGGAGGAGTGTCTTAAGATTCGTTCGGTTTTGGCTGAATTCGAGGAGCTCAACGTTGAGCACGTCAACCCTTACGCCCCTGAGCTTAAGGACGGCACTCCCAAGGAGAAGCGCTACCCTGTTGCCTTCCTTGGTGCTCGTGAATACATTTTCTCGGAGAACTCTGGTGTTCTTGGTGACGTTGCTGCTGGTAAGGAACAGACTTTCGGTACTTTGTTTGCTCGTACTTTGGCTCAGATTGGCGGTAAGTTGCACTACGGTCACCCCGATTTCTTGAACGCCACGTTCATGTTGACTCGTGGTGGTGTGTCCAAGGCCCAGAAGGGTTTGCATCTTAACGAAGATATTTATGCCGGTATGAACGCCATGTTGCGTGGTGGTAAGATTAAGCACTGCGAGTACTACCAGTGTGGTAAGGGTCGTGATATGGGTTTCGGTTCCATTTTGAACTTTACCACCAAGATTGGTGCTGGTATGGGTGAACAGATGTTGTCGCGTGAGTACTACTACCTTTCTACGCAGTTGCCCCTTGACCGTTTCCTTTCGTTCTACTACGGTCACCCTGGTTTCCACATTAACAACTTGTTCATTCAGATGTCTTTGCAGGTCTTCATTTTGGTGTTGGCTAACCTTAACTCGTTGGCCCACGAGCTGATTATTTGTTCTTACAACAAGGACTTGCCCGTGACCGATTTGTTGTACCCCCTTGGCTGCTACAACTTGGTTCCCGCTGTTGACTGGATTCGTCGTTACACCCTTTCCATTTTCATTGTGTTCTTCATTTCGTTTATTCCCTTGACGGTGCAAGAATTGATTGAGCGTGGTGTGTGGAAGGCGTTCCAGCGTTTCGTCCGTCACTTGATCTCGTTGTCGCCCATGTTTGAGGTTTTCGTTGCTCAGATTTACTCGTCTTCGATTGCTACCGACATGACTGTGGGTGGTGCCCGGTATATCTCCACTGGTCGTGGTTTCGCTACCCTGCGTATCCCCTTCTCGATTTTGTACCTGCGGTTCGCTGACCTGTCTATTTACATGGGTGCTCGTCTTATGTTGATTTTGTTCTTTGGTTCCGTGGCTCACTGGCAGGCTCCCTTGTTGTGGTTCTGGGCCTGTTTGACTGCGTTGATCATGTCTCCTTTCATTTTCAACCCCCACCAGTTTGCTTGGGAGGACTTCTTCATTGACTACCGTGACTTCATTCGCTGGTTGTCTCGTGGTAACACAAAGTGGCACCGCAACTCTTGGATTGGCTACGTGCGTCTCTCGCGGTCGCGTGTCACTGGTTTCAAGCGCAAGTTGCTCAACGATGTTTCTGAGAAGCTGGCTGGTGATGCTCTGCGTGCTCACCGTTCTAACGTCTTCATGGCGTCGATCTTGCCCACTGCCGTCTACACCGCTGGTTTGTTTGTTGCCTACACTTTCATCAACGCTCAGACTGGTGTCACCGAGTACGTGGACAAGGACGGTAACACTGCTCCTGCTGGTCCCGTCAACTCGGTCTTGCGTCTTATTATCTGTGCGCTTGGTCCGGTTGTGATCAACGCTGGTGTTGTCGGTGGCTGTTTTGGTTTGGCTTGTTGCGCTGGTCCCATGTTGGGCATGTGCTGCAAGAAGACCGGTGCCGTCATGGCTGGTATTGCTCACGGTGTTGCTGTTGTCATTCACATTGTGTTCTTCATTGTGATGTGGGTGATGGAGGGCTTCAACTTTGCTCGTATGTTGCTTGGTTTGGCCACCTGTATTTACGTGCAGCGTCTCTTGTTCAAGGTTCTTACTGTGTTGTGTCTTACCCGTGAGTTCAAGAACGACCGTGCCAACACGGCTTTCTGGACCGGTAAGTGGTACAACACCGGTATGGGCTGGATGGCGGTGACCCAGCCTGCTCGTGAATACGTTGCTAAGATTGTGGAGATGTCGGAGTTCGCTGGTGACTTTATGTTGGCTCACCTCATCTTGTTTGTCCAGTTGCCCATCTTGTTCATTCCCCTCATTGACAGATGGCACCTGACCATGTTGTTCTGGTTGAAGCCCCTGCAGTTGATCCGTCCTCCCATCTACTCGCTTAAGCAGGCCCGCTTGCGGAAGCGTATGGTGCGCAAGTACTGTACTTTATACTTCGCTGTCCTCGTGTTGTTCATCGTCATTATTGTCGCCCCCGCGGTGGCCCTGTCTCACATTGACAACATCGGTGACAAGTTGGAGAACATCGATGATGGTGGCATTGGGTTGTCTGGCTTGTTCCAGCCCCGTAACACTCTGAACAACGACACTGGCCCCAACATGCCCTCGGGTGGCCTGTGGTTGTCGCTGGCTTGGTCTTTCAATTCGATCAAGTTCACCGGCACCCTTTCGTCGTACACTACCAAGGAGTTCAAGTGA

Amino Acid Sequence（1883aa）

MSYNDNHHNYYDPNAPEGADGYYQAPYDGGQGPQEYYDPNMQYQQGAPEMEGGYDQGYHQQGQYGGEAFLDFSYGGQGGYDQYNGTQYTPLQMSYGGDPRSLGALTPIYGGQGQYDPTQFQMLLNLPYPAWLADPQAPIKVEHIEDIFIDLANKFGFQRDLMRNMFDYFMTLLDLRSLRMLPAQALLLLHADYIGGENANYRKWYFASQQDLDDSVGYANMQLGKIGRKARKAKRKLKKARQAAEEIGQDVDALDNELEGDYSLEAAEIRWKAKMNALLPEERVRDIALYLLMWGEANQIRFTPECLCFLFKLAMDYLMSPACQQRTEPVPEGDYLNRVITPLYRFLRSQVYEIYEGRFVKREKDHNKVIGYDDVNQLFWYPEGILRIIFEDGTRLVDMPQAERWGRLGEVEWNNVFFKTYKEIRTWLHFVTNFNRIWIIHGTIYWMYTAYNSPTLYTQNYVQTMNQQPLALSRWASAAIGGILASAIQIFATLFEWMFVPREWAGAQHLSRRLGFLVLILLLNLVPVVYTFYWAGLLSYSKLAHAVSIVGFFIAIATLLFYAVMPLGGLFTLYMNKSLRKYLASQTFTANFIKLRGIDMWMSYLLWVLVFLAKLVESYFFLTLSLRDPVRNLLTMTMRCIGELWWGFTLCRQQAKVVLGLMYAVDLLLFFLDTYMWYIICNCIFSIGRSFYLGISILTPWRNIFTRLPKRIYSKILATTEMEIKYKPKVLISQIWNAIVISMYREHLLAIDHVQKLLYHQVPSEIEGKRTLRAPTFFVLQDDNNFETEFFPRNSEAERRILFFAQSLATPILEPLPVDNMPTFTVFTPHYSEKILLSLREIIREDDQYSRVTLLEYLKQLHPVEWDCFVKDTKILAEETAAYENGDEEKASEDDGLKSKIDDLPFYCIGFKLAAPEYTLRTRIWASLRSQTLYRTVSGFMNYARAIKLLYRVENPELVQYFGGDPEGLELALEKMARRKFRFLVLMQRMAKFKDDEMENAEFLLRAYPDLQIAYLDEEPAMNEEEEPRVYSALIDGHCEMLENGRRRPKFRVQLSGNPILGDGKSDNQNHAIIFHRGEYIQLIDANQDNYLEECLKIRSVLAEFEELNVEHVNPYAPELKDGTPKEKRYPVAFLGAREYIFSENSGVLGDVAAGKEQTFGTLFARTLAQIGGKLHYGHPDFLNATFMLTRGGVSKAQKGLHLNEDIYAGMNAMLRGGKIKHCEYYQCGKGRDMGFGSILNFTTKIGAGMGEQMLSREYYYLSTQLPLDRFLSFYYGHPGFHINNLFIQMSLQVFILVLANLNSLAHELIICSYNKDLPVTDLLYPLGCYNLVPAVDWIRRYTLSIFIVFFISFIPLTVQELIERGVWKAFQRFVRHLISLSPMFEVFVAQIYSSSIATDMTVGGARYISTGRGFATLRIPFSILYLRFADLSIYMGARLMLILFFGSVAHWQAPLLWFWACLTALIMSPFIFNPHQFAWEDFFIDYRDFIRWLSRGNTKWHRNSWIGYVRLSRSRVTGFKRKLLNDVSEKLAGDALRAHRSNVFMASILPTAVYTAGLFVAYTFINAQTGVTEYVDKDGNTAPAGPVNSVLRLIICALGPVVINAGVVGGCFGLACCAGPMLGMCCKKTGAVMAGIAHGVAVVIHIVFFIVMWVMEGFNFARMLLGLATCIYVQRLLFKVLTVLCLTREFKNDRANTAFWTGKWYNTGMGWMAVTQPAREYVAKIVEMSEFAGDFMLAHLILFVQLPILFIPLIDRWHLTMLFWLKPLQLIRPPIYSLKQARLRKRMVRKYCTLYFAVLVLFIVIIVAPAVALSHIDNIGDKLENIDDGGIGLSGLFQPRNTLNNDTGPNMPSGGLWLSLAWSFNSIKFTGTLSSYTTKEFK*

3.*D. catenulata* Strain 10H1065 (T1861A[F621I]mutation)

Gene sequence（5652bp）

ATGTCGTACAACGATAACCATCATAATTATTATGACCCAAATGCCCCCGAGGGGGCAGATGGGTACTACCAGGCCCCGTATGACGGTGGTCAGGGCCCCCAGGAGTACTACGACCCCAACATGCAGTACCAGCAAGGGGCGCCCGAGATGGAAGGTGGCTACGACCAGGGTTACCACCAGCAGGGCCAGTACGGTGGTGAGGCGTTCCTGGATTTCAGCTACGGCGGCCAGGGCGGGTACGACCAGTACAACGGCACCCAGTACACCCCGCTGCAGATGAGTTACGGTGGGGACCCGCGGTCGCTGGGGGCGCTGACGCCAATCTACGGTGGCCAGGGCCAGTACGACCCTACCCAGTTCCAGATGCTGCTGAACTTGCCGTACCCGGCGTGGCTGGCTGACCCCCAGGCCCCGATCAAGGTGGAGCACATTGAGGACATTTTCATTGACCTTGCCAACAAATTTGGGTTCCAGCGGGACCTGATGCGTAACATGTTCGACTACTTCATGACGTTGTTGGACCTGCGGTCGCTGCGGATGCTGCCGGCGCAGGCGCTTTTGCTGTTGCACGCCGACTACATTGGTGGTGAGAACGCCAACTACCGCAAGTGGTACTTCGCTTCGCAGCAGGACTTGGACGACTCGGTGGGCTACGCCAACATGCAGTTGGGTAAGATTGGTCGCAAGGCCCGCAAGGCTAAGCGGAAGCTGAAGAAGGCTCGTCAGGCTGCCGAAGAGATTGGCCAAGACGTTGACGCTCTCGACAACGAGTTGGAGGGCGACTACTCTTTGGAGGCGGCCGAGATCCGCTGGAAGGCCAAGATGAATGCGTTGCTGCCCGAGGAGCGTGTTCGTGACATTGCCCTTTACTTATTGATGTGGGGTGAGGCCAACCAGATCCGTTTTACTCCCGAGTGCTTGTGTTTCTTGTTCAAGCTGGCGATGGACTACTTGATGTCGCCCGCGTGCCAGCAGCGTACCGAGCCTGTGCCTGAGGGTGACTACCTTAACCGGGTCATCACTCCTCTTTACCGGTTTTTGCGGTCGCAGGTGTACGAAATCTACGAGGGCCGGTTCGTCAAGCGTGAGAAGGACCACAACAAGGTGATTGGTTACGACGACGTTAACCAGTTGTTCTGGTACCCCGAGGGTATCCTGCGTATCATTTTTGAGGATGGTACCCGACTCGTCGATATGCCTCAAGCCGAGCGGTGGGGCCGCTTGGGTGAGGTTGAGTGGAACAACGTTTTCTTCAAGACCTACAAGGAGATTCGTACCTGGTTGCATTTCGTCACCAACTTTAACCGTATTTGGATTATCCACGGTACCATCTACTGGATGTACACGGCCTACAACTCTCCCACTTTGTACACCCAGAACTATGTGCAGACCATGAACCAGCAGCCGTTGGCGCTGTCTCGTTGGGCATCTGCCGCTATCGGTGGTATTCTTGCCTCCGCCATTCAGATTTTTGCTACCCTCTTCGAGTGGATGTTCGTTCCTCGTGAATGGGCGGGTGCTCAGCACCTTTCGCGCCGGTTGGGTTTCCTTGTGTTGATTTTGTTGTTGAACTTGGTGCCCGTTGTTTACACTTTCTACTGGGCCGGTTTGCTGTCTTACTCGAAGCTGGCGCACGCTGTGTCGATTGTCGGTTTCTTCATTGCCATCGCTACCCTTTTGTTCTACGCGGTTATGCCTTTGGGTGGCTTGTTCACCCTGTACATGAACAAGTCGCTGCGTAAGTACTTGGCGTCGCAGACTTTCACTGCCAACTTTATCAAGTTGCGTGGCATTGACATGTGGATGTCCTACCTTTTGTGGGTGCTCGTTTTCCTTGCCAAGCTCGTTGAATCGTACTTCATCCTTACCCTTTCGTTGCGTGACCCTGTTCGTAACTTGCTGACGATGACGATGCGGTGCATTGGTGAGCTGTGGTGGGGGTTCACCCTCTGTCGCCAGCAGGCCAAGGTTGTGTTGGGTTTGATGTACGCCGTCGACCTTTTGTTGTTCTTCCTTGATACCTACATGTGGTACATTATCTGTAACTGTATCTTCTCGATTGGTCGCTCGTTCTACCTCGGTATCTCCATTTTGACTCCTTGGCGTAACATCTTCACTCGGTTGCCCAAGCGTATCTACTCTAAGATCCTTGCCACCACCGAGATGGAGATCAAGTACAAGCCCAAGGTGTTGATCTCGCAGATTTGGAACGCTATTGTCATCTCCATGTACCGTGAGCACTTGTTGGCCATTGACCACGTTCAGAAGTTGTTGTACCACCAGGTGCCGTCCGAGATTGAGGGCAAGCGCACTCTCCGTGCACCCACTTTCTTCGTGCTGCAGGACGACAACAACTTTGAGACCGAGTTCTTCCCCCGTAACTCTGAAGCCGAGCGTCGTATTCTGTTCTTTGCTCAGTCGTTGGCTACCCCCATCTTGGAGCCTTTGCCCGTGGACAACATGCCCACTTTCACTGTGTTTACTCCTCATTACTCTGAAAAGATTTTGTTGTCTTTGCGGGAGATTATTCGTGAGGATGACCAATACTCGCGGGTGACCCTTTTGGAGTACTTGAAGCAGTTGCACCCTGTCGAGTGGGACTGTTTCGTTAAGGACACCAAGATTTTGGCTGAGGAGACTGCTGCTTACGAGAACGGTGACGAGGAGAAGGCTTCCGAAGATGACGGACTCAAGTCCAAGATCGACGATTTGCCCTTCTACTGCATTGGTTTCAAGCTGGCTGCCCCGGAGTACACTCTTCGTACTCGTATTTGGGCTTCGTTGCGTTCGCAGACTTTGTACCGTACTGTTTCTGGTTTCATGAACTACGCCCGTGCCATTAAGTTGTTGTACCGGGTGGAGAACCCTGAACTTGTCCAGTACTTTGGTGGTGACCCCGAGGGTCTCGAATTGGCTTTGGAAAAGATGGCTCGTCGTAAGTTCCGCTTCCTTGTGCTGATGCAGCGTATGGCTAAGTTTAAGGACGACGAGATGGAGAACGCTGAGTTCTTGTTGCGTGCCTACCCCGACTTGCAGATTGCCTACCTCGACGAGGAGCCGGCGATGAACGAGGAAGAGGAGCCGCGGGTGTACTCGGCTTTGATTGATGGTCACTGCGAGATGCTCGAAAACGGCCGTCGTCGTCCTAAGTTCCGGGTCCAGTTGTCGGGTAACCCCATTCTCGGTGACGGTAAGTCTGATAACCAGAACCATGCCATTATTTTCCACCGTGGTGAGTACATCCAGTTGATTGACGCTAACCAGGACAACTACTTGGAGGAGTGTCTTAAGATTCGTTCGGTTTTGGCTGAATTCGAGGAGCTCAACGTTGAGCACGTCAACCCTTACGCCCCTGAGCTTAAGGACAGCACTCCCAAGGAGAAGCGCTACCCTGTTGCCTTCCTTGGTGCTCGTGAATACATTTTCTCGGAGAACTCTGGTGTTCTTGGTGACGTTGCTGCTGGTAAGGAACAGACTTTCGGTACTTTGTTTGCTCGTACTTTGGCTCAGATTGGCGGTAAGTTGCACTACGGTCACCCCGATTTCTTGAACGCCACGTTCATGTTGACTCGTGGTGGTGTGTCCAAGGCCCAGAAGGGTTTGCATCTTAACGAAGATATTTATGCCGGTATGAACGCCATGTTGCGTGGTGGTAAGATTAAGCACTGCGAGTACTACCAGTGTGGTAAGGGTCGTGATATGGGTTTCGGTTCCATTTTGAACTTTACCACCAAGATTGGTGCTGGTATGGGTGAACAGATGTTGTCGCGTGAGTACTACTACCTTTCTACGCAGTTGCCCCTTGACCGTTTCCTTTCGTTCTACTACGGTCACCCTGGTTTCCACATTAACAACTTGTTCATTCAGATGTCTTTGCAGGTCTTCATTTTGGTGTTGGCTAACCTTAACTCGTTGGCCCACGAGCTGATTATTTGTTCTTACAACAAGGACTTGCCCGTGACCGATTTGTTGTACCCCCTTGGCTGCTACAACTTGGTTCCCGCTGTTGACTGGATTCGTCGTTACACCCTTTCCATTTTCATTGTGTTCTTCATTTCGTTTATTCCCTTGACGGTGCAAGAATTGATTGAGCGTGGTGTGTGGAAGGCGTTCCAGCGTTTCGTCCGTCACTTGATCTCGTTGTCGCCCATGTTTGAGGTTTTCGTTGCTCAGATTTACTCGTCTTCGATTGCTACCGACATGACTGTGGGTGGTGCCCGGTATATCTCGACTGGTCGTGGTTTCGCTACCCTGCGTATCCCCTTCTCGATTTTGTACCTGCGGTTCGCTGACCTGTCTATTTACATGGGTGCTCGTCTTATGTTGATTTTGTTCTTTGGTTCCGTGGCTCACTGGCAGGCTCCCTTGTTGTGGTTCTGGGCCTGTTTGACTGCGTTGATCATGTCTCCTTTCATTTTCAACCCCCACCAGTTTGCTTGGGAGGACTTCTTCATTGACTACCGTGACTTCATTCGCTGGTTGTCTCGTGGTAACACAAAGTGGCACCGCAACTCTTGGATTGGCTACGTGCGTCTCTCGCGGTCGCGTGTCACTGGTTTCAAGCGCAAGTTGCTCAACGATGTTTCTGAGAAGCTGGCTGGTGATGCTCTGCGTGCTCACCGTTCTAACGTCTTCATGGCGTCGATCTTGCCCACTGCCGTCTACACCGCTGGTTTGTTTGTTGCCTACACTTTCATCAACGCCCAGACTGGTGTCACCGAGTACGTGGACAAGGACGGTAACACTGCTCCTGCTGGTCCCGTCAACTCGGTCTTGCGTCTTATTATCTGTGCGCTTGGTCCGGTTGTGATCAACGCTGGTGTTGTCGGTGGCTGTTTTGGTTTGGCTTGTTGCGCTGGTCCCATGTTGGGCATGTGCTGCAAGAAGACCGGTGCCGTCATGGCTGGTATTGCTCACGGTGTTGCTGTTGTCATTCACATTGTGTTCTTCATTGTGATGTGGGTGATGGAGGGCTTTAACTTTGCTCGTATGTTGCTTGGTTTGGCCACCTGTATTTACGTGCAGCGTCTCTTGTTCAAGGTTCTTACTGTGTTGTGTCTTACCCGTGAGTTCAAGAACGACCGTGCTAACACGGCTTTCTGGACCGGTAAGTGGTACAACACCGGTATGGGCTGGATGGCGGTGACCCAGCCTGCTCGTGAATACGTTGCTAAGATTGTGGAGATGTCGGAGTTCGCTGGTGACTTTATGTTGGCTCACCTCATCTTGTTTGTCCAGTTGCCCATCTTGTTCATTCCCCTCATTGACAGATGGCACCTGACCATGTTGTTCTGGTTGAAGCCCCTGCAGTTGATCCGTCCTCCCATCTACTCGCTTAAGCAGGCCCGCTTGCGGAAGCGTATGGTGCGCAAGTACTGTACTTTATACTTCGCTGTCCTCGTGTTGTTCATCGTCATTATTGTCGCCCCCGCGGTGGCCCTGTCTCACATTGACAACATCGGTGACAAGTTGGAGAACATCGATGATGGTGGCATTGGGTTGTCTGGCTTGTTCCAGCCCCGTAACACTCTGAACAACGACACTGGCCCCAACATGCCCTCGGGTGGCCTGTGGTTGTCGCTGGCTTGGTCTTTCAATTCGATCAAGTTCACCGGCACCCTTTCGTCGTACACTACCAAGGAGTTCAAGTGA

Amino Acid Sequence（1883aa）

MSYNDNHHNYYDPNAPEGADGYYQAPYDGGQGPQEYYDPNMQYQQGAPEMEGGYDQGYHQQGQYGGEAFLDFSYGGQGGYDQYNGTQYTPLQMSYGGDPRSLGALTPIYGGQGQYDPTQFQMLLNLPYPAWLADPQAPIKVEHIEDIFIDLANKFGFQRDLMRNMFDYFMTLLDLRSLRMLPAQALLLLHADYIGGENANYRKWYFASQQDLDDSVGYANMQLGKIGRKARKAKRKLKKARQAAEEIGQDVDALDNELEGDYSLEAAEIRWKAKMNALLPEERVRDIALYLLMWGEANQIRFTPECLCFLFKLAMDYLMSPACQQRTEPVPEGDYLNRVITPLYRFLRSQVYEIYEGRFVKREKDHNKVIGYDDVNQLFWYPEGILRIIFEDGTRLVDMPQAERWGRLGEVEWNNVFFKTYKEIRTWLHFVTNFNRIWIIHGTIYWMYTAYNSPTLYTQNYVQTMNQQPLALSRWASAAIGGILASAIQIFATLFEWMFVPREWAGAQHLSRRLGFLVLILLLNLVPVVYTFYWAGLLSYSKLAHAVSIVGFFIAIATLLFYAVMPLGGLFTLYMNKSLRKYLASQTFTANFIKLRGIDMWMSYLLWVLVFLAKLVESYFILTLSLRDPVRNLLTMTMRCIGELWWGFTLCRQQAKVVLGLMYAVDLLLFFLDTYMWYIICNCIFSIGRSFYLGISILTPWRNIFTRLPKRIYSKILATTEMEIKYKPKVLISQIWNAIVISMYREHLLAIDHVQKLLYHQVPSEIEGKRTLRAPTFFVLQDDNNFETEFFPRNSEAERRILFFAQSLATPILEPLPVDNMPTFTVFTPHYSEKILLSLREIIREDDQYSRVTLLEYLKQLHPVEWDCFVKDTKILAEETAAYENGDEEKASEDDGLKSKIDDLPFYCIGFKLAAPEYTLRTRIWASLRSQTLYRTVSGFMNYARAIKLLYRVENPELVQYFGGDPEGLELALEKMARRKFRFLVLMQRMAKFKDDEMENAEFLLRAYPDLQIAYLDEEPAMNEEEEPRVYSALIDGHCEMLENGRRRPKFRVQLSGNPILGDGKSDNQNHAIIFHRGEYIQLIDANQDNYLEECLKIRSVLAEFEELNVEHVNPYAPELKDSTPKEKRYPVAFLGAREYIFSENSGVLGDVAAGKEQTFGTLFARTLAQIGGKLHYGHPDFLNATFMLTRGGVSKAQKGLHLNEDIYAGMNAMLRGGKIKHCEYYQCGKGRDMGFGSILNFTTKIGAGMGEQMLSREYYYLSTQLPLDRFLSFYYGHPGFHINNLFIQMSLQVFILVLANLNSLAHELIICSYNKDLPVTDLLYPLGCYNLVPAVDWIRRYTLSIFIVFFISFIPLTVQELIERGVWKAFQRFVRHLISLSPMFEVFVAQIYSSSIATDMTVGGARYISTGRGFATLRIPFSILYLRFADLSIYMGARLMLILFFGSVAHWQAPLLWFWACLTALIMSPFIFNPHQFAWEDFFIDYRDFIRWLSRGNTKWHRNSWIGYVRLSRSRVTGFKRKLLNDVSEKLAGDALRAHRSNVFMASILPTAVYTAGLFVAYTFINAQTGVTEYVDKDGNTAPAGPVNSVLRLIICALGPVVINAGVVGGCFGLACCAGPMLGMCCKKTGAVMAGIAHGVAVVIHIVFFIVMWVMEGFNFARMLLGLATCIYVQRLLFKVLTVLCLTREFKNDRANTAFWTGKWYNTGMGWMAVTQPAREYVAKIVEMSEFAGDFMLAHLILFVQLPILFIPLIDRWHLTMLFWLKPLQLIRPPIYSLKQARLRKRMVRKYCTLYFAVLVLFIVIIVAPAVALSHIDNIGDKLENIDDGGIGLSGLFQPRNTLNNDTGPNMPSGGLWLSLAWSFNSIKFTGTLSSYTTKEFK*

4.*D. catenulata* Strain 16HLJ6019(T4043G[I1348S]mutation)

Gene sequence（5652bp）

ATGTCGTACAACGATAACCATCATAATTATTATGACCCAAATGCCCCCGAGGGGGCAGATGGGTACTACCAGGCCCCGTATGACGGTGGTCAGGGCCCCCAGGAGTACTACGACCCCAACATGCAGTACCAGCAAGGGGCGCCCGAGATGGAAGGTGGCTACGACCAGGGTTACCACCAGCAGGGCCAGTACGGTGGTGAGGCGTTCCTGGATTTCAGCTACGGCGGCCAGGGCGGGTACGACCAGTACAACGGCACCCAGTACACCCCGCTGCAGATGAGTTACGGTGGGGACCCGCGGTCGCTGGGGGCGCTGACGCCAATCTACGGTGGCCAGGGCCAGTACGACCCTACCCAGTTCCAGATGCTGCTGAACTTGCCGTACCCGGCGTGGCTGGCTGACCCCCAGGCCCCGATCAAGGTGGAGCACATTGAGGACATTTTCATTGACCTTGCCAACAAATTTGGGTTCCAGCGGGACCTGATGCGTAACATGTTCGACTACTTCATGACGTTGTTGGACCTGCGGTCGCTGCGGATGCTGCCGGCGCAGGCGCTTTTGCTGTTGCACGCCGACTACATTGGTGGTGAGAACGCCAACTACCGCAAGTGGTACTTCGCTTCGCAGCAGGACTTGGACGACTCGGTGGGCTACGCCAACATGCAGTTGGGTAAGATTGGTCGCAAGGCCCGCAAGGCTAAGCGGAAGCTGAAGAAGGCTCGTCAGGCTGCCGAAGAGATTGGCCAAGACGTTGACGCTCTCGACAACGAGTTGGAGGGCGACTACTCTTTGGAGGCGGCCGAGATCCGCTGGAAGGCCAAGATGAATGCGTTGCTGCCCGAGGAGCGTGTTCGTGACATTGCCCTTTACTTATTGATGTGGGGTGAGGCCAACCAGATCCGTTTTACTCCCGAGTGCTTGTGTTTCTTGTTCAAGCTGGCGATGGACTACTTGATGTCGCCCGCGTGCCAGCAGCGTACCGAGCCTGTGCCTGAGGGTGACTACCTTAACCGGGTCATCACTCCTCTTTACCGGTTTTTGCGGTCGCAGGTGTACGAAATCTACGAGGGCCGGTTCGTCAAGCGTGAGAAGGACCACAACAAGGTGATTGGTTACGACGACGTTAACCAGTTGTTCTGGTACCCCGAGGGTATCCTGCGTATCATTTTTGAGGATGGTACCCGACTCGTCGATATGCCTCAAGCCGAGCGGTGGGGCCGCTTGGGTGAGGTTGAGTGGAACAACGTTTTCTTCAAGACCTACAAGGAGATTCGTACCTGGTTGCATTTCGTCACCAACTTTAACCGTATTTGGATTATCCACGGTACCATCTACTGGATGTACACGGCCTACAACTCTCCCACTTTGTACACCCAGAACTATGTGCAGACCATGAACCAGCAGCCGTTGGCGCTGTCTCGTTGGGCATCTGCCGCTATCGGTGGTATTCTTGCCTCCGCCATTCAGATTTTTGCTACCCTCTTCGAGTGGATGTTCGTTCCTCGTGAATGGGCGGGTGCTCAGCACCTTTCGCGCCGGTTGGGTTTCCTTGTGTTGATTTTGTTGTTGAACTTGGTGCCCGTTGTTTACACTTTCTACTGGGCCGGTTTGCTGTCTTACTCGAAGCTGGCGCACGCTGTGTCGATTGTCGGTTTCTTCATTGCCATCGCTACCCTTTTGTTCTACGCGGTTATGCCTTTGGGTGGCTTGTTCACCCTGTACATGAACAAGTCGCTGCGTAAGTACTTGGCGTCGCAGACTTTCACTGCCAACTTTATCAAGTTGCGTGGCATTGACATGTGGATGTCCTACCTTTTGTGGGTGCTCGTTTTCCTTGCCAAGCTCGTTGAATCGTACTTCTTCCTTACCCTTTCGTTGCGTGACCCTGTTCGTAACTTGCTGACGATGACGATGCGGTGCATTGGTGAGCTGTGGTGGGGGTTCACCCTCTGTCGCCAGCAGGCCAAGGTTGTGTTGGGTTTGATGTACGCCGTCGACCTTTTGTTGTTCTTCCTTGATACCTACATGTGGTACATTATCTGTAACTGTATCTTCTCGATTGGTCGCTCGTTCTACCTCGGTATCTCCATTTTGACTCCTTGGCGTAACATCTTCACTCGGTTGCCCAAGCGTATCTACTCTAAGATCCTTGCCACCACCGAGATGGAGATCAAGTACAAGCCCAAGGTGTTGATCTCGCAGATTTGGAACGCTATTGTCATCTCCATGTACCGTGAGCACTTGTTGGCCATTGACCACGTTCAGAAGTTGTTGTACCACCAGGTGCCGTCCGAGATTGAGGGCAAGCGCACTCTCCGTGCACCCACTTTCTTCGTGCTGCAGGACGACAACAACTTTGAGACCGAGTTCTTCCCCCGTAACTCTGAAGCCGAGCGTCGTATTCTGTTCTTTGCTCAGTCGTTGGCTACCCCCATCTTGGAGCCTTTGCCCGTGGACAACATGCCCACTTTCACTGTGTTTACTCCTCATTACTCTGAAAAGATTTTGTTGTCTTTGCGGGAGATTATTCGTGAGGATGACCAATACTCGCGGGTGACCCTTTTGGAGTACTTGAAGCAGTTGCACCCTGTCGAGTGGGACTGTTTCGTTAAGGACACCAAGATTTTGGCTGAGGAGACTGCTGCTTACGAGAACGGTGACGAGGAGAAGGCTTCCGAAGATGACGGACTCAAGTCCAAGATCGACGATTTGCCCTTCTACTGCATTGGTTTCAAGCTGGCTGCCCCGGAGTACACTCTTCGTACTCGTATTTGGGCTTCGTTGCGTTCGCAGACTTTGTACCGTACTGTTTCTGGTTTCATGAACTACGCCCGTGCCATTAAGTTGTTGTACCGGGTGGAGAACCCTGAACTTGTCCAGTACTTTGGTGGTGACCCCGAGGGTCTCGAATTGGCTTTGGAAAAGATGGCTCGTCGTAAGTTCCGCTTCCTTGTGCTGATGCAGCGTATGGCTAAGTTTAAGGACGACGAGATGGAGAACGCTGAGTTCTTGTTGCGTGCCTACCCCGACTTGCAGATTGCCTACCTCGACGAGGAGCCGGCGATGAACGAGGAAGAGGAGCCGCGGGTGTACTCGGCTTTGATTGATGGTCACTGCGAGATGCTCGAAAACGGCCGTCGTCGTCCTAAGTTCCGGGTCCAGTTGTCGGGTAACCCCATTCTCGGTGACGGTAAGTCTGATAACCAGAACCATGCCATTATTTTCCACCGTGGTGAGTACATCCAGTTGATTGACGCTAACCAGGACAACTACTTGGAGGAGTGTCTTAAGATTCGTTCGGTTTTGGCTGAATTCGAGGAGCTCAACGTTGAGCACGTCAACCCTTACGCCCCTGAGCTTAAGGACAGCACTCCCAAGGAGAAGCGCTACCCTGTTGCCTTCCTTGGTGCTCGTGAATACATTTTCTCGGAGAACTCTGGTGTTCTTGGTGACGTTGCTGCTGGTAAGGAACAGACTTTCGGTACTTTGTTTGCTCGTACTTTGGCTCAGATTGGCGGTAAGTTGCACTACGGTCACCCCGATTTCTTGAACGCCACGTTCATGTTGACTCGTGGTGGTGTGTCCAAGGCCCAGAAGGGTTTGCATCTTAACGAAGATATTTATGCCGGTATGAACGCCATGTTGCGTGGTGGTAAGATTAAGCACTGCGAGTACTACCAGTGTGGTAAGGGTCGTGATATGGGTTTCGGTTCCATTTTGAACTTTACCACCAAGATTGGTGCTGGTATGGGTGAACAGATGTTGTCGCGTGAGTACTACTACCTTTCTACGCAGTTGCCCCTTGACCGTTTCCTTTCGTTCTACTACGGTCACCCTGGTTTCCACATTAACAACTTGTTCATTCAGATGTCTTTGCAGGTCTTCATTTTGGTGTTGGCTAACCTTAACTCGTTGGCCCACGAGCTGATTATTTGTTCTTACAACAAGGACTTGCCCGTGACCGATTTGTTGTACCCCCTTGGCTGCTACAACTTGGTTCCCGCTGTTGACTGGATTCGTCGTTACACCCTTTCCATTTTCAGTGTGTTCTTCATTTCGTTTATTCCCTTGACGGTGCAAGAATTGATTGAGCGTGGTGTGTGGAAGGCGTTCCAGCGTTTCGTCCGTCACTTGATCTCGTTGTCGCCCATGTTTGAGGTTTTCGTTGCTCAGATTTACTCGTCTTCGATTGCTACCGACATGACTGTGGGTGGTGCCCGGTATATCTCGACTGGTCGTGGTTTCGCTACCCTGCGTATCCCCTTCTCGATTTTGTACCTGCGGTTCGCTGACCTGTCTATTTACATGGGTGCTCGTCTTATGTTGATTTTGTTCTTTGGTTCCGTGGCTCACTGGCAGGCTCCCTTGTTGTGGTTCTGGGCCTGTTTGACTGCGTTGATCATGTCTCCTTTCATTTTCAACCCCCACCAGTTTGCTTGGGAGGACTTCTTCATTGACTACCGTGACTTCATTCGCTGGTTGTCTCGTGGTAACACAAAGTGGCACCGCAACTCTTGGATTGGCTACGTGCGTCTCTCGCGGTCGCGTGTCACTGGTTTCAAGCGCAAGTTGCTCAACGATGTTTCTGAGAAGCTGGCTGGTGATGCTCTGCGTGCTCACCGTTCTAACGTCTTCATGGCGTCGATCTTGCCCACTGCCGTCTACACCGCTGGTTTGTTTGTTGCCTACACTTTCATCAACGCCCAGACTGGTGTCACCGAGTACGTGGACAAGGACGGTAACACTGCTCCTGCTGGTCCCGTCAACTCGGTCTTGCGTCTTATTATCTGTGCGCTTGGTCCGGTTGTGATCAACGCTGGTGTTGTCGGTGGCTGTTTTGGTTTGGCTTGTTGCGCTGGTCCCATGTTGGGCATGTGCTGCAAGAAGACCGGTGCCGTCATGGCTGGTATTGCTCACGGTGTTGCTGTTGTCATTCACATTGTGTTCTTCATTGTGATGTGGGTGATGGAGGGCTTTAACTTTGCTCGTATGTTGCTTGGTTTGGCCACCTGTATTTACGTGCAGCGTCTCTTGTTCAAGGTTCTTACTGTGTTGTGTCTTACCCGTGAGTTCAAGAACGACCGTGCTAACACGGCTTTCTGGACCGGTAAGTGGTACAACACCGGTATGGGCTGGATGGCGGTGACCCAGCCTGCTCGTGAATACGTTGCTAAGATTGTGGAGATGTCGGAGTTCGCTGGTGACTTTATGTTGGCTCACCTCATCTTGTTTGTCCAGTTGCCCATCTTGTTCATTCCCCTCATTGACAGATGGCACCTGACCATGTTGTTCTGGTTGAAGCCCCTGCAGTTGATCCGTCCTCCCATCTACTCGCTTAAGCAGGCCCGCTTGCGGAAGCGTATGGTGCGCAAGTACTGTACTTTATACTTCGCTGTCCTCGTGTTGTTCATCGTCATTATTGTCGCCCCCGCGGTGGCCCTGTCTCACATTGACAACATCGGTGACAAGTTGGAGAACATCGATGATGGTGGCATTGGGTTGTCTGGCTTGTTCCAGCCCCGTAACACTCTGAACAACGACACTGGCCCCAACATGCCCTCGGGTGGCCTGTGGTTGTCGCTGGCTTGGTCTTTCAATTCGATCAAGTTCACCGGCACCCTTTCGTCGTACACTACCAAGGAGTTCAAGTGA

Amino Acid Sequence（1883aa）

MSYNDNHHNYYDPNAPEGADGYYQAPYDGGQGPQEYYDPNMQYQQGAPEMEGGYDQGYHQQGQYGGEAFLDFSYGGQGGYDQYNGTQYTPLQMSYGGDPRSLGALTPIYGGQGQYDPTQFQMLLNLPYPAWLADPQAPIKVEHIEDIFIDLANKFGFQRDLMRNMFDYFMTLLDLRSLRMLPAQALLLLHADYIGGENANYRKWYFASQQDLDDSVGYANMQLGKIGRKARKAKRKLKKARQAAEEIGQDVDALDNELEGDYSLEAAEIRWKAKMNALLPEERVRDIALYLLMWGEANQIRFTPECLCFLFKLAMDYLMSPACQQRTEPVPEGDYLNRVITPLYRFLRSQVYEIYEGRFVKREKDHNKVIGYDDVNQLFWYPEGILRIIFEDGTRLVDMPQAERWGRLGEVEWNNVFFKTYKEIRTWLHFVTNFNRIWIIHGTIYWMYTAYNSPTLYTQNYVQTMNQQPLALSRWASAAIGGILASAIQIFATLFEWMFVPREWAGAQHLSRRLGFLVLILLLNLVPVVYTFYWAGLLSYSKLAHAVSIVGFFIAIATLLFYAVMPLGGLFTLYMNKSLRKYLASQTFTANFIKLRGIDMWMSYLLWVLVFLAKLVESYFFLTLSLRDPVRNLLTMTMRCIGELWWGFTLCRQQAKVVLGLMYAVDLLLFFLDTYMWYIICNCIFSIGRSFYLGISILTPWRNIFTRLPKRIYSKILATTEMEIKYKPKVLISQIWNAIVISMYREHLLAIDHVQKLLYHQVPSEIEGKRTLRAPTFFVLQDDNNFETEFFPRNSEAERRILFFAQSLATPILEPLPVDNMPTFTVFTPHYSEKILLSLREIIREDDQYSRVTLLEYLKQLHPVEWDCFVKDTKILAEETAAYENGDEEKASEDDGLKSKIDDLPFYCIGFKLAAPEYTLRTRIWASLRSQTLYRTVSGFMNYARAIKLLYRVENPELVQYFGGDPEGLELALEKMARRKFRFLVLMQRMAKFKDDEMENAEFLLRAYPDLQIAYLDEEPAMNEEEEPRVYSALIDGHCEMLENGRRRPKFRVQLSGNPILGDGKSDNQNHAIIFHRGEYIQLIDANQDNYLEECLKIRSVLAEFEELNVEHVNPYAPELKDSTPKEKRYPVAFLGAREYIFSENSGVLGDVAAGKEQTFGTLFARTLAQIGGKLHYGHPDFLNATFMLTRGGVSKAQKGLHLNEDIYAGMNAMLRGGKIKHCEYYQCGKGRDMGFGSILNFTTKIGAGMGEQMLSREYYYLSTQLPLDRFLSFYYGHPGFHINNLFIQMSLQVFILVLANLNSLAHELIICSYNKDLPVTDLLYPLGCYNLVPAVDWIRRYTLSIFSVFFISFIPLTVQELIERGVWKAFQRFVRHLISLSPMFEVFVAQIYSSSIATDMTVGGARYISTGRGFATLRIPFSILYLRFADLSIYMGARLMLILFFGSVAHWQAPLLWFWACLTALIMSPFIFNPHQFAWEDFFIDYRDFIRWLSRGNTKWHRNSWIGYVRLSRSRVTGFKRKLLNDVSEKLAGDALRAHRSNVFMASILPTAVYTAGLFVAYTFINAQTGVTEYVDKDGNTAPAGPVNSVLRLIICALGPVVINAGVVGGCFGLACCAGPMLGMCCKKTGAVMAGIAHGVAVVIHIVFFIVMWVMEGFNFARMLLGLATCIYVQRLLFKVLTVLCLTREFKNDRANTAFWTGKWYNTGMGWMAVTQPAREYVAKIVEMSEFAGDFMLAHLILFVQLPILFIPLIDRWHLTMLFWLKPLQLIRPPIYSLKQARLRKRMVRKYCTLYFAVLVLFIVIIVAPAVALSHIDNIGDKLENIDDGGIGLSGLFQPRNTLNNDTGPNMPSGGLWLSLAWSFNSIKFTGTLSSYTTKEFK*

5.*D. catenulata* Strain 17TJ970 (C1874T[S625L]+A3367G[S1123G]+T4062G[F1354L]mutation)

Gene sequence（5652bp）

ATGTCGTACAACGATAACCATCATAATTATTATGACCCAAATGCCCCCGAGGGGGCAGATGGGTACTACCAGGCCCCGTATGACGGTGGTCAGGGCCCCCAGGAGTACTACGACCCCAACATGCAGTACCAGCAAGGGGCGCCCGAGATGGAAGGTGGCTACGACCAGGGTTACCACCAGCAGGGCCAGTACGGTGGTGAGGCGTTCCTGGATTTCAGCTACGGCGGCCAGGGCGGGTACGACCAGTACAACGGCACCCAGTACACCCCGCTGCAGATGAGTTACGGTGGGGACCCGCGGTCGCTGGGGGCGCTGACGCCAATCTACGGTGGCCAGGGCCAGTACGACCCTACCCAGTTCCAGATGCTGCTGAACTTGCCGTACCCGGCGTGGCTGGCTGACCCCCAGGCCCCGATCAAGGTGGAGCACATTGAGGACATTTTCATTGACCTTGCCAACAAATTTGGGTTCCAGCGGGACCTGATGCGTAACATGTTCGACTACTTCATGACGTTGTTGGACCTGCGGTCGCTGCGGATGCTGCCGGCGCAGGCGCTTTTGCTGTTGCACGCCGACTACATTGGTGGTGAGAACGCCAACTACCGCAAGTGGTACTTCGCTTCGCAGCAGGACTTGGACGACTCGGTGGGCTACGCCAACATGCAGTTGGGTAAGATTGGTCGCAAGGCCCGCAAGGCTAAGCGGAAGCTGAAGAAGGCTCGTCAGGCTGCCGAAGAGATTGGCCAAGACGTTGACGCTCTCGACAACGAGTTGGAGGGCGACTACTCTTTGGAGGCGGCCGAGATCCGCTGGAAGGCCAAGATGAATGCGTTGCTGCCCGAGGAGCGTGTTCGTGACATTGCCCTTTACTTATTGATGTGGGGTGAGGCCAACCAGATCCGTTTTACTCCCGAGTGCTTGTGTTTCTTGTTCAAGCTGGCGATGGACTACTTGATGTCGCCCGCGTGCCAGCAGCGTACCGAGCCTGTGCCTGAGGGTGACTACCTTAACCGGGTCATCACTCCTCTTTACCGGTTTTTGCGGTCGCAGGTGTACGAAATCTACGAGGGCCGGTTCGTCAAGCGTGAGAAGGACCACAACAAGGTGATTGGTTACGACGACGTTAACCAGTTGTTCTGGTACCCCGAGGGTATCCTGCGTATCATTTTTGAGGATGGTACCCGACTCGTCGACATGCCTCAGGCCGAGCGGTGGGGCCGCTTGGGTGAGGTTGAGTGGAACAACGTTTTCTTCAAGACCTACAAGGAGATTCGTACCTGGTTGCATTTCGTCACCAACTTTAACCGTATTTGGATTATCCACGGTACCATCTACTGGATGTACACGGCCTACAACTCTCCCACTTTGTACACCCAGAACTATGTGCAGACCATGAACCAGCAGCCGTTGGCGCTGTCTCGTTGGGCATCTGCCGCTATCGGTGGTATTCTTGCCTCCGCCATTCAGATTTTTGCTACCCTCTTCGAGTGGATGTTCGTTCCTCGTGAATGGGCGGGTGCTCAGCACCTTTCGCGCCGGTTGGGTTTCCTTGTATTGATTTTGTTGTTGAACTTGGTGCCCGTTGTTTACACTTTCTACTGGGCCGGTTTGCTGTCTTACTCGAAGCTGGCGCACGCTGTGTCGATTGTCGGTTTCTTCATTGCCATCGCTACCCTTTTGTTCTACGCGGTTATGCCTTTGGGTGGCTTGTTCACCCTGTACATGAACAAGTCGCTGCGTAAGTACTTGGCGTCGCAGACTTTCACTGCCAACTTTATCAAGTTGCGTGGCATTGACATGTGGATGTCCTACCTTTTGTGGGTGCTCGTTTTCCTTGCCAAGCTCGTTGAATCGTACTTCTTCCTTACCCTTTTGTTGCGTGACCCTGTTCGTAACTTGCTGACGATGACGATGCGGTGCATTGGTGAGCTGTGGTGGGGGTTCACCCTCTGTCGCCAGCAGGCCAAGGTTGTGTTGGGTTTGATGTACGCCGTCGACCTTTTGTTGTTCTTCCTTGATACCTACATGTGGTACATTATCTGTAACTGTATCTTCTCGATTGGTCGCTCGTTCTACCTCGGTATCTCCATTTTGACTCCCTGGCGTAACATCTTCACTCGGTTGCCCAAGCGTATCTACTCTAAGATCCTTGCCACCACCGAGATGGAGATCAAGTACAAGCCCAAGGTGTTGATCTCGCAGATTTGGAACGCTATTGTCATCTCCATGTACCGTGAGCACTTGTTGGCCATTGACCACGTTCAGAAGTTGTTGTACCACCAGGTGCCGTCCGAGATTGAGGGCAAGCGCACTCTCCGTGCACCCACTTTCTTCGTGCTGCAGGACGACAACAACTTTGAGACCGAGTTCTTCCCCCGTAACTCTGAAGCCGAGCGTCGTATTCTGTTCTTTGCTCAGTCGTTGGCTACCCCCATCTTGGAGCCTTTGCCCGTGGACAACATGCCCACTTTCACTGTGTTTACTCCTCATTACTCTGAAAAGATTTTGTTGTCTTTGCGGGAGATTATTCGTGAGGATGACCAATACTCGCGGGTGACCCTTTTGGAGTACTTGAAGCAGTTGCACCCTGTCGAGTGGGACTGTTTCGTTAAGGACACCAAGATTTTGGCTGAGGAGACTGCTGCTTACGAGAACGGTGACGAGGAGAAGGCTTCCGAAGATGACGGACTCAAGTCCAAGATCGACGATTTGCCCTTCTACTGCATTGGTTTCAAGCTGGCTGCCCCGGAGTACACTCTTCGTACTCGTATTTGGGCTTCCTTGCGTTCGCAGACTTTGTACCGTACTGTTTCTGGTTTCATGAACTACGCCCGTGCCATTAAGTTGTTGTACCGGGTGGAGAACCCTGAGCTTGTCCAGTACTTTGGTGGTGACCCCGAGGGTCTCGAATTGGCTTTGGAAAAGATGGCTCGTCGTAAGTTCCGCTTCCTTGTGCTGATGCAGCGTATGGCTAAGTTTAAGGACGACGAGATGGAGAACGCTGAGTTCTTGTTGCGTGCCTACCCCGACTTGCAAATTGCCTACCTCGACGAGGAGCCGGCGATGAACGAGGAAGAGGAGCCGCGGGTGTACTCGGCTTTGATTGATGGTCACTGCGAGATGCTCGAAAACGGCCGTCGTCGTCCTAAGTTCCGGGTCCAGTTGTCGGGTAACCCCATTCTCGGTGACGGTAAGTCTGATAACCAGAACCATGCCATTATTTTCCACCGTGGTGAGTACATCCAGTTGATTGACGCTAACCAGGACAACTACTTGGAGGAGTGTCTTAAGATTCGTTCGGTTTTGGCTGAATTCGAGGAGCTCAACGTTGAGCACGTCAACCCTTACGCCCCTGAGCTTAAGGACGGCACTCCCAAGGAGAAGCGCTACCCTGTTGCCTTCCTTGGTGCTCGTGAATACATTTTCTCGGAGAACTCTGGTGTTCTTGGTGACGTTGCTGCTGGTAAGGAACAGACTTTCGGTACTTTGTTTGCTCGTACTTTGGCTCAGATTGGCGGTAAGTTGCACTACGGTCACCCCGATTTCTTGAACGCCACGTTCATGTTGACTCGTGGTGGTGTGTCCAAGGCCCAGAAGGGTTTGCATCTTAACGAAGATATTTATGCCGGTATGAACGCCATGTTGCGTGGTGGTAAGATTAAGCACTGCGAGTACTACCAGTGTGGTAAGGGTCGTGATATGGGTTTCGGTTCCATTTTGAACTTTACCACCAAGATTGGTGCTGGTATGGGTGAACAGATGTTGTCGCGTGAGTACTACTACCTTTCTACGCAGTTGCCCCTTGACCGTTTCCTTTCGTTCTACTACGGTCACCCTGGTTTCCACATTAACAACTTGTTCATTCAGATGTCTTTGCAGGTCTTCATTTTGGTGTTGGCTAACCTTAACTCGTTGGCCCACGAGCTGATTATTTGTTCTTACAACAAGGACTTGCCCGTGACCGATTTGTTGTACCCCCTTGGCTGCTACAACTTGGTTCCCGCTGTTGACTGGATTCGTCGTTACACCCTTTCCATTTTCATTGTGTTCTTCATTTCGTTGATTCCCTTGACGGTGCAAGAATTGATTGAGCGTGGTGTGTGGAAGGCGTTCCAGCGTTTCGTCCGTCACTTGATCTCGTTGTCGCCCATGTTTGAGGTTTTCGTTGCTCAGATTTACTCGTCTTCGATTGCTACCGACATGACTGTGGGTGGTGCCCGGTATATCTCCACTGGTCGTGGTTTCGCTACCCTGCGTATCCCCTTCTCGATTTTGTACCTGCGGTTCGCTGACCTGTCTATTTACATGGGTGCTCGTCTTATGTTGATTTTGTTCTTTGGTTCCGTGGCTCACTGGCAGGCTCCCTTGTTGTGGTTCTGGGCCTGTTTGACTGCGTTGATCATGTCTCCTTTCATTTTCAACCCCCACCAGTTTGCTTGGGAGGACTTCTTCATTGACTACCGTGACTTCATTCGCTGGTTGTCTCGTGGTAACACAAAGTGGCACCGCAACTCTTGGATTGGCTACGTGCGTCTCTCGCGGTCGCGTGTCACTGGTTTCAAGCGCAAGTTGCTCAACGATGTTTCTGAGAAGCTGGCTGGTGATGCTCTGCGTGCTCACCGTTCTAACGTCTTCATGGCGTCGATCTTGCCCACTGCCGTCTACACCGCTGGTTTGTTTGTTGCCTACACTTTCATCAACGCTCAGACTGGTGTCACCGAGTACGTGGACAAGGACGGTAACACTGCTCCTGCTGGTCCCGTCAACTCGGTCTTGCGTCTTATTATCTGTGCGCTTGGTCCGGTTGTGATCAACGCTGGTGTTGTCGGTGGCTGTTTTGGTTTGGCTTGTTGCGCTGGTCCCATGTTGGGCATGTGCTGCAAGAAGACCGGTGCCGTCATGGCTGGTATTGCTCACGGTGTTGCTGTTGTCATTCACATTGTGTTCTTCATTGTGATGTGGGTGATGGAGGGCTTCAACTTTGCTCGTATGTTGCTTGGTTTGGCCACCTGTATTTACGTGCAGCGTCTCTTGTTCAAGGTTCTTACTGTGTTGTGTCTTACCCGTGAGTTCAAGAACGACCGTGCCAACACGGCTTTCTGGACCGGTAAGTGGTACAACACCGGTATGGGCTGGATGGCGGTGACCCAGCCTGCTCGTGAATACGTTGCTAAGATTGTGGAGATGTCGGAGTTCGCTGGTGACTTTATGTTGGCTCACCTCATCTTGTTTGTCCAGTTGCCCATCTTGTTCATTCCCCTCATTGACAGATGGCACCTGACCATGTTGTTCTGGTTGAAGCCCCTGCAGTTGATCCGTCCTCCCATCTACTCGCTTAAGCAGGCCCGCTTGCGGAAGCGTATGGTGCGCAAGTACTGTACTTTATACTTCGCTGTCCTCGTGTTGTTCATCGTCATTATTGTCGCCCCCGCGGTGGCCCTGTCTCACATTGACAACATCGGTGACAAGTTGGAGAACATCGATGATGGTGGCATTGGGTTGTCTGGCTTGTTCCAGCCCCGTAACACTCTGAACAACGACACTGGCCCCAACATGCCCTCGGGTGGCCTGTGGTTGTCGCTGGCTTGGTCTTTCAATTCGATCAAGTTCACCGGCACCCTTTCGTCGTACACTACCAAGGAGTTCAAGTGA

Amino Acid Sequence（1883aa）

MSYNDNHHNYYDPNAPEGADGYYQAPYDGGQGPQEYYDPNMQYQQGAPEMEGGYDQGYHQQGQYGGEAFLDFSYGGQGGYDQYNGTQYTPLQMSYGGDPRSLGALTPIYGGQGQYDPTQFQMLLNLPYPAWLADPQAPIKVEHIEDIFIDLANKFGFQRDLMRNMFDYFMTLLDLRSLRMLPAQALLLLHADYIGGENANYRKWYFASQQDLDDSVGYANMQLGKIGRKARKAKRKLKKARQAAEEIGQDVDALDNELEGDYSLEAAEIRWKAKMNALLPEERVRDIALYLLMWGEANQIRFTPECLCFLFKLAMDYLMSPACQQRTEPVPEGDYLNRVITPLYRFLRSQVYEIYEGRFVKREKDHNKVIGYDDVNQLFWYPEGILRIIFEDGTRLVDMPQAERWGRLGEVEWNNVFFKTYKEIRTWLHFVTNFNRIWIIHGTIYWMYTAYNSPTLYTQNYVQTMNQQPLALSRWASAAIGGILASAIQIFATLFEWMFVPREWAGAQHLSRRLGFLVLILLLNLVPVVYTFYWAGLLSYSKLAHAVSIVGFFIAIATLLFYAVMPLGGLFTLYMNKSLRKYLASQTFTANFIKLRGIDMWMSYLLWVLVFLAKLVESYFFLTLLLRDPVRNLLTMTMRCIGELWWGFTLCRQQAKVVLGLMYAVDLLLFFLDTYMWYIICNCIFSIGRSFYLGISILTPWRNIFTRLPKRIYSKILATTEMEIKYKPKVLISQIWNAIVISMYREHLLAIDHVQKLLYHQVPSEIEGKRTLRAPTFFVLQDDNNFETEFFPRNSEAERRILFFAQSLATPILEPLPVDNMPTFTVFTPHYSEKILLSLREIIREDDQYSRVTLLEYLKQLHPVEWDCFVKDTKILAEETAAYENGDEEKASEDDGLKSKIDDLPFYCIGFKLAAPEYTLRTRIWASLRSQTLYRTVSGFMNYARAIKLLYRVENPELVQYFGGDPEGLELALEKMARRKFRFLVLMQRMAKFKDDEMENAEFLLRAYPDLQIAYLDEEPAMNEEEEPRVYSALIDGHCEMLENGRRRPKFRVQLSGNPILGDGKSDNQNHAIIFHRGEYIQLIDANQDNYLEECLKIRSVLAEFEELNVEHVNPYAPELKDGTPKEKRYPVAFLGAREYIFSENSGVLGDVAAGKEQTFGTLFARTLAQIGGKLHYGHPDFLNATFMLTRGGVSKAQKGLHLNEDIYAGMNAMLRGGKIKHCEYYQCGKGRDMGFGSILNFTTKIGAGMGEQMLSREYYYLSTQLPLDRFLSFYYGHPGFHINNLFIQMSLQVFILVLANLNSLAHELIICSYNKDLPVTDLLYPLGCYNLVPAVDWIRRYTLSIFIVFFISLIPLTVQELIERGVWKAFQRFVRHLISLSPMFEVFVAQIYSSSIATDMTVGGARYISTGRGFATLRIPFSILYLRFADLSIYMGARLMLILFFGSVAHWQAPLLWFWACLTALIMSPFIFNPHQFAWEDFFIDYRDFIRWLSRGNTKWHRNSWIGYVRLSRSRVTGFKRKLLNDVSEKLAGDALRAHRSNVFMASILPTAVYTAGLFVAYTFINAQTGVTEYVDKDGNTAPAGPVNSVLRLIICALGPVVINAGVVGGCFGLACCAGPMLGMCCKKTGAVMAGIAHGVAVVIHIVFFIVMWVMEGFNFARMLLGLATCIYVQRLLFKVLTVLCLTREFKNDRANTAFWTGKWYNTGMGWMAVTQPAREYVAKIVEMSEFAGDFMLAHLILFVQLPILFIPLIDRWHLTMLFWLKPLQLIRPPIYSLKQARLRKRMVRKYCTLYFAVLVLFIVIIVAPAVALSHIDNIGDKLENIDDGGIGLSGLFQPRNTLNNDTGPNMPSGGLWLSLAWSFNSIKFTGTLSSYTTKEFK*

**(C)**

Plasmid pYES2/CT gene sequence（5963bp）

ACGGATTAGAAGCCGCCGAGCGGGTGACAGCCCTCCGAAGGAAGACTCTCCTCCGTGCGTCCTCGTCTTCACCGGTCGCGTTCCTGAAACGCAGATGTGCCTCGCGCCGCACTGCTCCGAACAATAAAGATTCTACAATACTAGCTTTTATGGTTATGAAGAGGAAAAATTGGCAGTAACCTGGCCCCACAAACCTTCAAATGAACGAATCAAATTAACAACCATAGGATGATAATGCGATTAGTTTTTTAGCCTTATTTCTGGGGTAATTAATCAGCGAAGCGATGATTTTTGATCTATTAACAGATATATAAATGCAAAAACTGCATAACCACTTTAACTAATACTTTCAACATTTTCGGTTTGTATTACTTCTTATTCAAATGTAATAAAAGTATCAACAAAAAATTGTTAATATACCTCTATACTTTAACGTCAAGGAGAAAAAACCCCGGATCGGACTACTAGCAGCTGTAATACGACTCACTATAGGGAATATTAAGCTTGGTACCGAGCTCGGATCCACTAGTAACGGCCGCCAGTGTGCTGGAATTCTGCAGATATCCAGCACAGTGGCGGCCGCTCGAGTCTAGAGGGCCCTTCGAAGGTAAGCCTATCCCTAACCCTCTCCTCGGTCTCGATTCTACGCGTACCGGTCATCATCACCATCACCATTGAGTTTAAACCCGCTGATCCTAGAGGGCCGCATCATGTAATTAGTTATGTCACGCTTACATTCACGCCCTCCCCCCACATCCGCTCTAACCGAAAAGGAAGGAGTTAGACAACCTGAAGTCTAGGTCCCTATTTATTTTTTTATAGTTATGTTAGTATTAAGAACGTTATTTATATTTCAAATTTTTCTTTTTTTTCTGTACAGACGCGTGTACGCATGTAACATTATACTGAAAACCTTGCTTGAGAAGGTTTTGGGACGCTCGAAGGCTTTAATTTGCAAGCTGCGGCCCTGCATTAATGAATCGGCCAACGCGCGGGGAGAGGCGGTTTGCGTATTGGGCGCTCTTCCGCTTCCTCGCTCACTGACTCGCTGCGCTCGGTCGTTCGGCTGCGGCGAGCGGTATCAGCTCACTCAAAGGCGGTAATACGGTTATCCACAGAATCAGGGGATAACGCAGGAAAGAACATGTGAGCAAAAGGCCAGCAAAAGCCCAGGAACCGTAAAAAGGCCGCGTTGCTGGCGTTTTTCCATAGGCTCCGCCCCCCTGACGAGCATCACAAAAATCGACGCTCAAGTCAGAGGTGGCGAAACCCGACAGGACTATAAAGATACCAGGCGTTTCCCCCTGGAAGCTCCCTCGTGCGCTCTCCTGTTCCGACCCTGCCGCTTACCGGATACCTGTCCGCCTTTCTCCCTTCGGGAAGCGTGGCGCTTTCTCATAGCTCACGCTGTAGGTATCTCAGTTCGGTGTAGGTCGTTCGCTCCAAGCTGGGCTGTGTGCACGAACCCCCCGTTCAGCCCGACCGCTGCGCCTTATCCGGTAACTATCGTCTTGAGTCCAACCCGGTAAGACACGACTTATCGCCACTGGCAGCAGCCACTGGTAACAGGATTAGCAGAGCGAGGTATGTAGGCGGTGCTACAGAGTTCTTGAAGTGGTGGCCTAACTACGGCTACACTAGAAGGACAGTATTTGGTATCTGCGCTCTGCTGAAGCCAGTTACCTTCGGAAAAAGAGTTGGTAGCTCTTGATCCGGCAAACAAACCACCGCTGGTAGCGGTGGTTTTTTTGTTTGCAAGCAGCAGATTACGCGCAGAAAAAAAGGATCTCAAGAAGATCCTTTGATCTTTTCTACGGGGTCTGACGCTCAGTGGAACGAAAACTCACGTTAAGGGATTTTGGTCATGAGATTATCAAAAAGGATCTTCACCTAGATCCTTTTAAATTAAAAATGAAGTTTTAAATCAATCTAAAGTATATATGAGTAAACTTGGTCTGACAGTTACCAATGCTTAATCAGTGAGGCACCTATCTCAGCGATCTGTCTATTTCGTTCATCCATAGTTGCCTGACTCCCCGTCGTGTAGATAACTACGATACGGGAGCGCTTACCATCTGGCCCCAGTGCTGCAATGATACCGCGAGACCCACGCTCACCGGCTCCAGATTTATCAGCAATAAACCAGCCAGCCGGAAGGGCCGAGCGCAGAAGTGGTCCTGCAACTTTATCCGCCTCCATCCAGTCTATTAATTGTTGCCGGGAAGCTAGAGTAAGTAGTTCGCCAGTTAATAGTTTGCGCAACGTTGTTGGCATTGCTACAGGCATCGTGGTGTCACTCTCGTCGTTTGGTATGGCTTCATTCAGCTCCGGTTCCCAACGATCAAGGCGAGTTACATGATCCCCCATGTTGTGCAAAAAAGCGGTTAGCTCCTTCGGTCCTCCGATCGTTGTCAGAAGTAAGTTGGCCGCAGTGTTATCACTCATGGTTATGGCAGCACTGCATAATTCTCTTACTGTCATGCCATCCGTAAGATGCTTTTCTGTGACTGGTGAGTACTCAACCAAGTCATTCTGAGAATAGTGTATGCGGCGACCGAGTTGCTCTTGCCCGGCGTCAATACGGGATAATAGTGTATCACATAGCAGAACTTTAAAAGTGCTCATCATTGGAAAACGTTCTTCGGGGCGAAAACTCTCAAGGATCTTACCGCTGTTGAGATCCAGTTCGATGTAACCCACTCGTGCACCCAACTGATCTTCAGCATCTTTTACTTTCACCAGCGTTTCTGGGTGAGCAAAAACAGGAAGGCAAAATGCCGCAAAAAAGGGAATAAGGGCGACACGGAAATGTTGAATACTCATACTCTTCCTTTTTCAATGGGTAATAACTGATATAATTAAATTGAAGCTCTAATTTGTGAGTTTAGTATACATGCATTTACTTATAATACAGTTTTTTAGTTTTGCTGGCCGCATCTTCTCAAATATGCTTCCCAGCCTGCTTTTCTGTAACGTTCACCCTCTACCTTAGCATCCCTTCCCTTTGCAAATAGTCCTCTTCCAACAATAATAATGTCAGATCCTGTAGAGACCACATCATCCACGGTTCTATACTGTTGACCCAATGCGTCTCCCTTGTCATCTAAACCCACACCGGGTGTCATAATCAACCAATCGTAACCTTCATCTCTTCCACCCATGTCTCTTTGAGCAATAAAGCCGATAACAAAATCTTTGTCGCTCTTCGCAATGTCAACAGTACCCTTAGTATATTCTCCAGTAGATAGGGAGCCCTTGCATGACAATTCTGCTAACATCAAAAGGCCTCTAGGTTCCTTTGTTACTTCTTCTGCCGCCTGCTTCAAACCGCTAACAATACCTGGGCCCACCACACCGTGTGCATTCGTAATGTCTGCCCATTCTGCTATTCTGTATACACCCGCAGAGTACTGCAATTTGACTGTATTACCAATGTCAGCAAATTTTCTGTCTTCGAAGAGTAAAAAATTGTACTTGGCGGATAATGCCTTTAGCGGCTTAACTGTGCCCTCCATGGAAAAATCAGTCAAGATATCCACATGTGTTTTTAGTAAACAAATTTTGGGACCTAATGCTTCAACTAACTCCAGTAATTCCTTGGTGGTACGAACATCCAATGAAGCACACAAGTTTGTTTGCTTTTCGTGCATGATATTAAATAGCTTGGCAGCAACAGGACTAGGATGAGTAGCAGCACGTTCCTTATATGTAGCTTTCGACATGATTTATCTTCGTTTCCTGCAGGTTTTTGTTCTGTGCAGTTGGGTTAAGAATACTGGGCAATTTCATGTTTCTTCAACACTACATATGCGTATATATACCAATCTAAGTCTGTGCTCCTTCCTTCGTTCTTCCTTCTGTTCGGAGATTACCGAATCAAAAAAATTTCAAAGAAACCGAAATCAAAAAAAAGAATAAAAAAAAAATGATGAATTGAATTGAAAAGCTAGCTTATCGATGATAAGCTGTCAAAGATGAGAATTAATTCCACGGACTATAGACTATACTAGATACTCCGTCTACTGTACGATACACTTCCGCTCAGGTCCTTGTCCTTTAACGAGGCCTTACCACTCTTTTGTTACTCTATTGATCCAGCTCAGCAAAGGCAGTGTGATCTAAGATTCTATCTTCGCGATGTAGTAAAACTAGCTAGACCGAGAAAGAGACTAGAAATGCAAAAGGCACTTCTACAATGGCTGCCATCATTATTATCCGATGTGACGCTGCAGCTTCTCAATGATATTCGAATACGCTTTGAGGAGATACAGCCTAATATCCGACAAACTGTTTTACAGATTTACGATCGTACTTGTTACCCATCATTGAATTTTGAACATCCGAACCTGGGAGTTTTCCCTGAAACAGATAGTATATTTGAACCTGTATAATAATATATAGTCTAGCGCTTTACGGAAGACAATGTATGTATTTCGGTTCCTGGAGAAACTATTGCATCTATTGCATAGGTAATCTTGCACGTCGCATCCCCGGTTCATTTTCTGCGTTTCCATCTTGCACTTCAATAGCATATCTTTGTTAACGAAGCATCTGTGCTTCATTTTGTAGAACAAAAATGCAACGCGAGAGCGCTAATTTTTCAAACAAAGAATCTGAGCTGCATTTTTACAGAACAGAAATGCAACGCGAAAGCGCTATTTTACCAACGAAGAATCTGTGCTTCATTTTTGTAAAACAAAAATGCAACGCGACGAGAGCGCTAATTTTTCAAACAAAGAATCTGAGCTGCATTTTTACAGAACAGAAATGCAACGCGAGAGCGCTATTTTACCAACAAAGAATCTATACTTCTTTTTTGTTCTACAAAAATGCATCCCGAGAGCGCTATTTTTCTAACAAAGCATCTTAGATTACTTTTTTTCTCCTTTGTGCGCTCTATAATGCAGTCTCTTGATAACTTTTTGCACTGTAGGTCCGTTAAGGTTAGAAGAAGGCTACTTTGGTGTCTATTTTCTCTTCCATAAAAAAAGCCTGACTCCACTTCCCGCGTTTACTGATTACTAGCGAAGCTGCGGGTGCATTTTTTCAAGATAAAGGCATCCCCGATTATATTCTATACCGATGTGGATTGCGCATACTTTGTGAACAGAAAGTGATAGCGTTGATGATTCTTCATTGGTCAGAAAATTATGAACGGTTTCTTCTATTTTGTCTCTATATACTACGTATAGGAAATGTTTACATTTTCGTATTGTTTTCGATTCACTCTATGAATAGTTCTTACTACAATTTTTTTGTCTAAAGAGTAATACTAGAGATAAACATAAAAAATGTAGAGGTCGAGTTTAGATGCAAGTTCAAGGAGCGAAAGGTGGATGGGTAGGTTATATAGGGATATAGCACAGAGATATATAGCAAAGAGATACTTTTGAGCAATGTTTGTGGAAGCGGTATTCGCAATGGGAAGCTCCACCCCGGTTGATAATCAGAAAAGCCCCAAAAACAGGAAGATTGTATAAGCAAATATTTAAATTGTAAACGTTAATATTTTGTTAAAATTCGCGTTAAATTTTTGTTAAATCAGCTCATTTTTTAACGAATAGCCCGAAATCGGCAAAATCCCTTATAAATCAAAAGAATAGACCGAGATAGGGTTGAGTGTTGTTCCAGTTTCCAACAAGAGTCCACTATTAAAGAACGTGGACTCCAACGTCAAAGGGCGAAAAAGGGTCTATCAGGGCGATGGCCCACTACGTGAACCATCACCCTAATCAAGTTTTTTGGGGTCGAGGTGCCGTAAAGCAGTAAATCGGAAGGGTAAACGGATGCCCCCATTTAGAGCTTGACGGGGAAAGCCGGCGAACGTGGCGAGAAAGGAAGGGAAGAAAGCGAAAGGAGCGGGGGCTAGGGCGGTGGGAAGTGTAGGGGTCACGCTGGGCGTAACCACCACACCCGCCGCGCTTAATGGGGCGCTACAGGGCGCGTGGGGATGATCCACTAGT

# Supplementary Figures and Tables

## Supplementary Figures


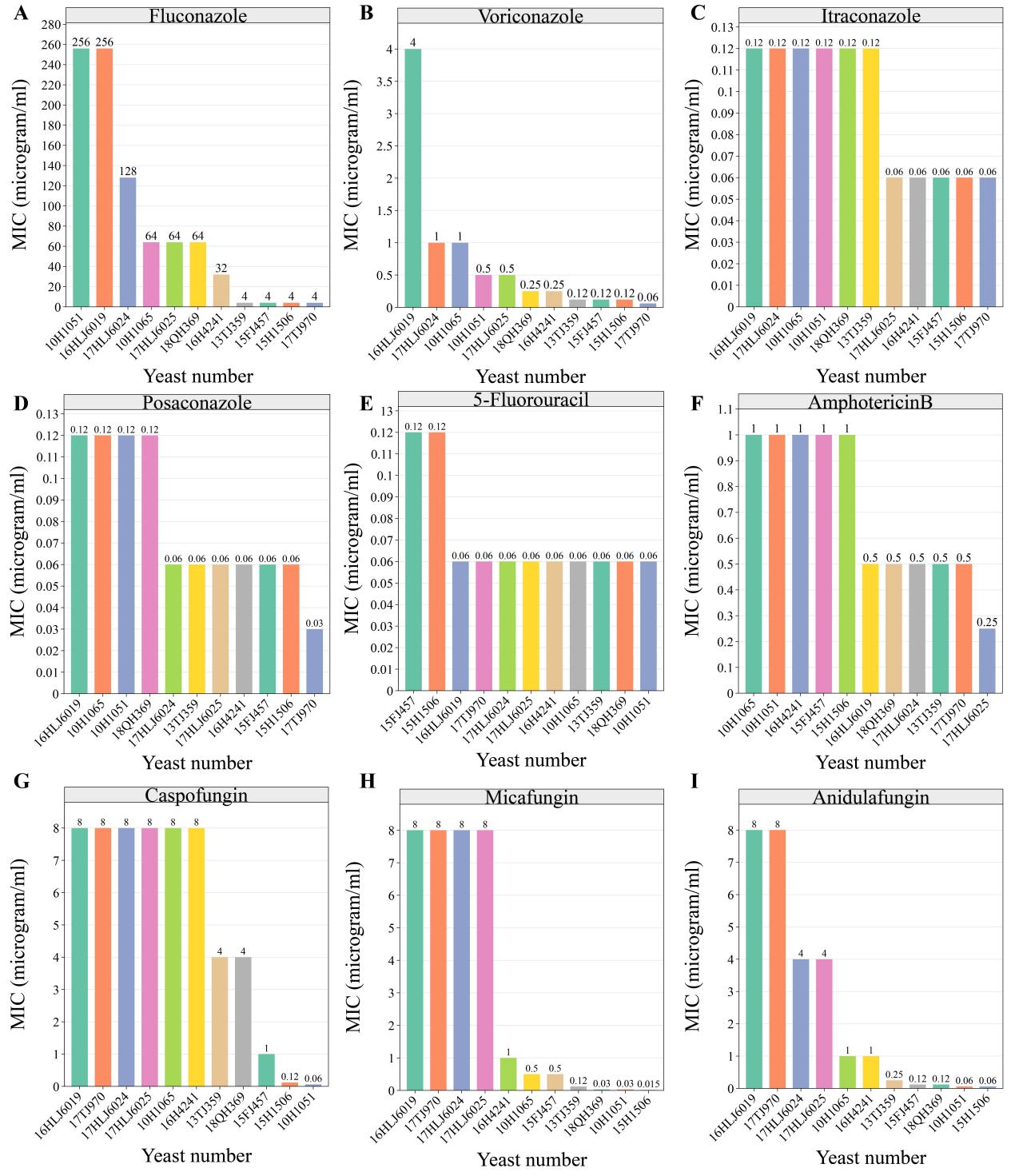


**Supplementary Figure 1.** Minimum inhibitory concentration (MIC) of 9 antifungal agents against 11 strains of *D. catenulata* .


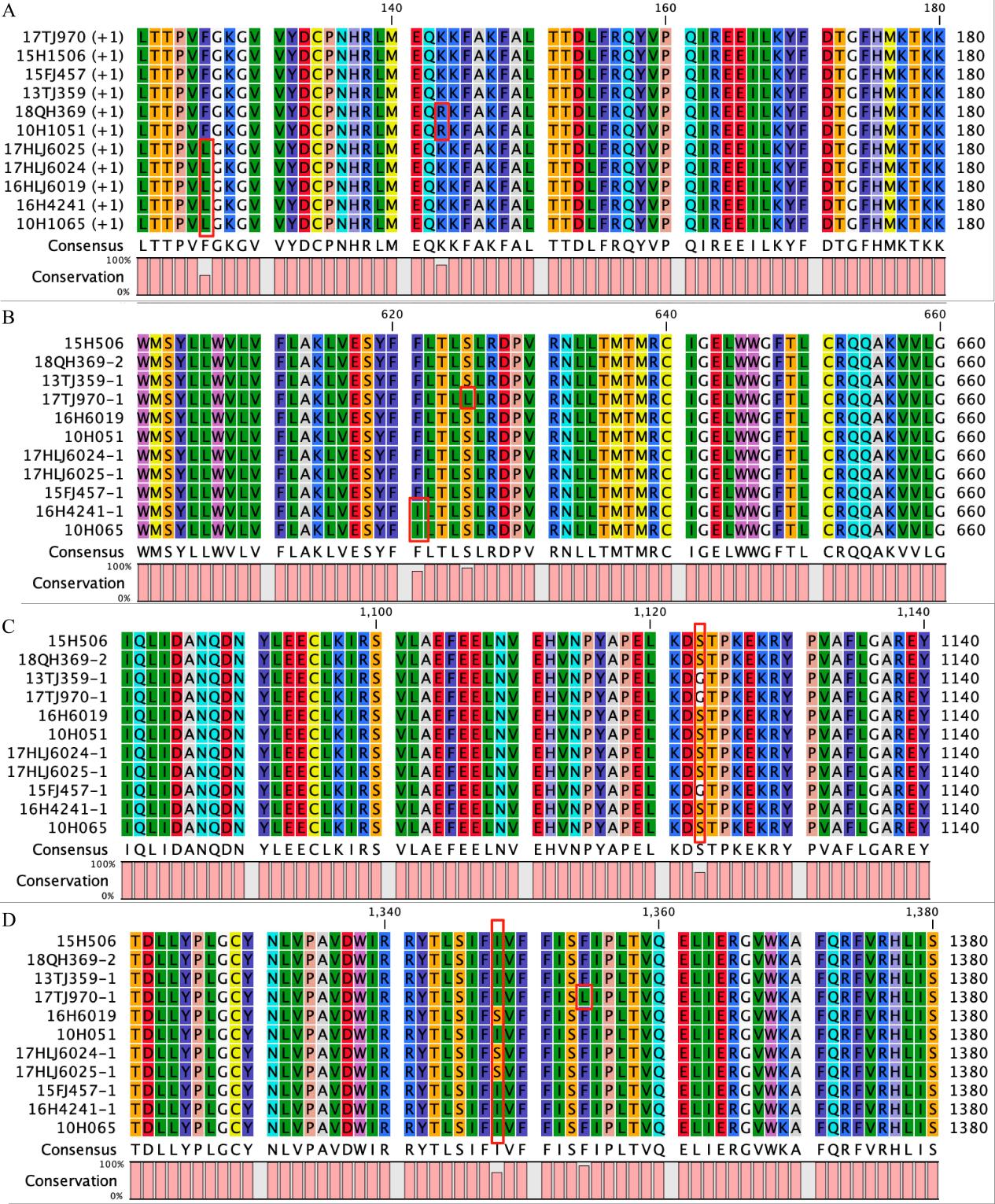


**Supplementary Figure 2.** Amino acid substitutions in 11 strains of *D. catenulata*: (A) Erg11 protein; (B–D) Fks1 protein.

**2.2 Supplementary Table 1.** Primer sequences for the *ERG11* and *FKS1* genes of *C. catenulata.*

| Gene | Number | Orientation | Sequence（5ʹ-3ʹ） |
| --- | --- | --- | --- |
| ERG11 | 1 | F | ACATTATTTATTGCCCCATG |
|  | 1 | R | GCAAGTATCCCGCTTTTCCC |
| FKS1 | 1 | F | GCACCCGATTTTCTACCTCA |
|  | 1 | R | GGTAAAGAGGAGTGATGACCC |
|  | 2 | F | GGAAGCTGAAGAAGGCTCGT |
|  | 2 | R | CTTACGCAGCGACTTGTTCAT |
|  | 3 | F | CCCAGAACTATGTGCAGACCA |
|  | 3 | R | CACGGGCAAAGGCTCCAAGAT |
|  | 4 | F | CGATTGGTCGCTCGTTCTAC |
|  | 4 | R | GCAAGGAAGCCCAAATACGAG |
|  | 5 | F | CTTTTGGAGTACTTGAAGCAG |
|  | 5 | R | TTAATCTTACCACCACGCAAC |
|  | 6 | F | TGCTGCTGGTAAGGAACAGAC |
|  | 6 | R | AAGAAGTCCTCCCAAGCAAAC |
|  | 7 | F | TCGTTGTCGCCCATGTTTGA |
|  | 7 | R | TCTTGAACTCACGGGTAAGA |
|  | 8 | F | TGTTGCGCTGGTCCCATGTT |
|  | 8 | R | CCCGGTCATCAACTCGTG |

**Supplementary Table 2.** Molecular Dynamics Simulation of Docking Effect between Drugs and Target Protein Molecules

| protein | protein system | binding energy (kcal/mol) | ΔΔG (vs. wild type) | weakening factor of binding affinity (-fold) | structure effect |
| --- | --- | --- | --- | --- | --- |
| Erg11 | Wild-type | -7.8 | 0.0 | 1.0 | Strong binding benchmark |
|  | F126L mutant | -7.5 | +0.3 | 1.7 | Expand the pocket capacity in combination  The amplitude of ligand oscillation increases  Loss of hydrophobic contact area |
|  | K143R mutant | -7.2 | +0.6 | 3.4 | Twisted hydrogen bond angle  Increased spatial hindrance |
| Fks1 | Wild-type | -9.4 | 0.0 | 1.0 | Strong binding benchmark |
|  | F621I | -9.0 | +0.4 | 2.3 | Hydrophobic volume loss |
|  | I1348S | -9.0 | +0.4 | 2.3 | Destroy hydrophobic pockets  Possible introduction of spatial conflicts |
|  | S1123G | -9.1 | +0.3 | 1.7 | Loss of hydrogen bonding ability  Conformational perturbation |
|  | S1123G+S625L+F1354L  Triple mutant | -8.3 | +1.1 | 6.4 | Significant weakening dominated by synergistic effects |

ΔΔG (binding free energy change) = Mutant binding energy - Wild type binding energy（Positive values indicate weakened binding）
